# Stereospecific induction of apoptosis in tumor cells via endogenous C_16_-ceramide and distinct transcripts

**DOI:** 10.1038/cddiscovery.2015.13

**Published:** 2015-07-27

**Authors:** M Blaess, HP Le, RA Claus, M Kohl, H-P Deigner

**Affiliations:** 1 Center for Sepsis Control and Care (CSCC), Jena University Hospital, Erlanger Allee 101, D-07747 Jena, Germany; 2 Clinic for Anaesthesiology and Intensive Care, Jena University Hospital, Erlanger Allee 101, D-07747 Jena, Germany; 3 Medical and Life Sciences Faculty, Furtwangen University, Jakob-Kienzle-Strasse 17, D-78054 Villingen-Schwenningen, Germany; 4 Fraunhofer Institute IZI, Leipzig, EXIM Department, Schillingallee 68, D-18057 Rostock, Germany

## Abstract

Concentration and distribution of individual endogenous ceramide species is crucial for apoptosis induction in response to various stimuli. Exogenous ceramide analogs induce apoptosis and can in turn modify the composition/concentrations of endogenous ceramide species and associated signaling. In this study, we show here that the elevation of endogenous C_16_-ceramide levels is a common feature of several known apoptosis-inducing triggers like mmLDL, TNF-alpha, H_2_O_2_ and exogenous C_6_-ceramide. *Vice versa* apoptosis requires elevation of endogenous C_16_-ceramide levels in cells. Enantiomers of a synthetic ceramide analog HPL-1RS36N have been developed as probes and vary in their capacity to inducing apoptosis in macrophages and HT-29 cells. Apoptosis induction by the two synthetic ceramide analogs HPL-39N and HPL-1R36N correlates with generation of cellular C_16_-ceramide concentration. In contrast to the S-enantiomer HPL-1S36N, the R-enantiomer HPL-1R36N shows significant effects on the expression of distinct genes known to be involved in cell cycle, cell growth and cell death (CXCL10, CCL5 and TNF-alpha), similarly on apoptosis induction. Enantioselective effects on transcription induced by metabolically stable synthetic probes provide clues on molecular mechanisms of ceramide-induced signaling, as well as leads for future anti-cancer agents.

## Introduction

Ceramides with fatty acids of varying chain length bound as amides are components of the sphingomyelin (SM) cycle and well-established signaling molecules, since activation of *sphingomyelinases* (*SMases*) and subsequent ceramide generation is involved in signal transduction of the cellular stress response, in particular during stress-induced apoptosis.^1–3^ The substrate of these pathways, SM, is an inert phospho-sphingolipid abundant in all eukaryotic cell types. On pro-inflammatory or pro-apoptotic stimulation, the SMases (phospholipase-like enzymes) catalyze its breakdown into ceramides which are highly bioactive lipid-mediators with two asymmetric carbons.^4,5^ These molecules function as common, converging messengers in apoptosis, and are also generated by signaling through *CD95-Fas/Apo1*, tumor necrosis factor alpha (*TNF-alpha*), ionizing radiation or chemotherapeutic agents.^6–8^ Moreover, in chronic atherosclerotic states, initiation of apoptosis following treatment of macrophages with oxidatively modified lipoproteins is mediated via an increase in the intracellular ceramide concentration.^9^

Elucidation of distinct steps of apoptosis initiated by synthetic, metabolically stabilized and more rigid analogs of ceramide with defined stereochemical configuration offers a valuable strategy to define structure-activity relationships and may foster the development of pro-apoptotic anti-cancer agents or agents for enhancing or retaining the potency of well-known cancer drugs.

## Results

### Effect of apoptogenic agents, C_6_-ceramide and synthetic, conformationally restricted ceramide analogs on C_16_- and C_24_-ceramides

**Figure 1:**
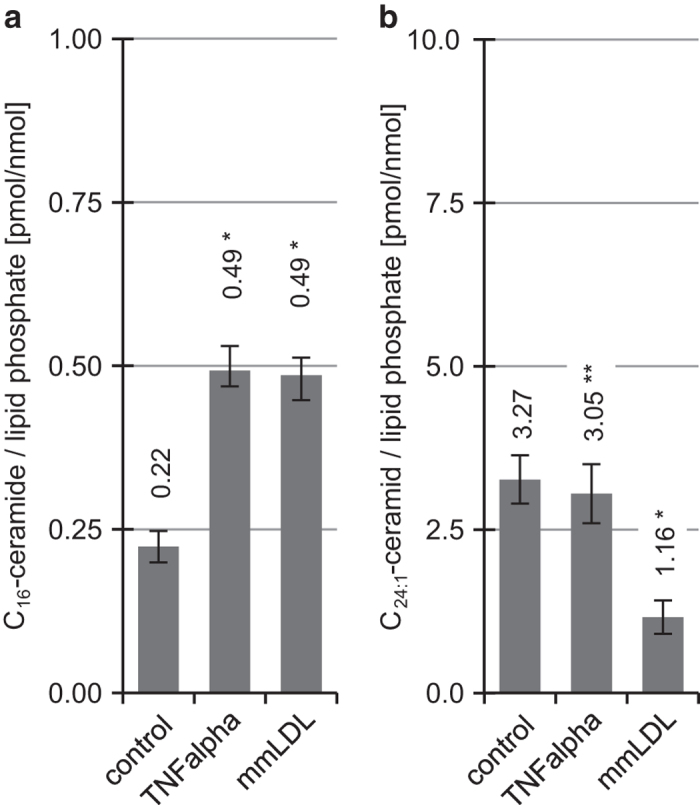
Effect of mmLDL (54 *μ*g ml^−1^) and *TNF-alpha* (3 ng ml^−1^) on ceramide concentration in macrophages. (**a**) C_16_-ceramide and (**b**) C_24:1_-ceramide concentrations in macrophages after 4 h treatment are shown as mean±S.E., *n*=3. One-way ANOVA in combination with *post hoc t*-tests and Bonferroni's correction for multiple testing, *P*<0.0001 (one-way ANOVA); significant with *P*<0.05: *versus control, ** mmLDL *versus* TNF-alpha.

We have previously demonstrated that exogenous C_6_-ceramide provokes an increase in total ceramide levels (determined by diacylglycerolkinase-assay in fibroblasts, an effect inhibitable with our agent NB06.^10,11^ Here we examined whether these effects accompany changes of endogenous C_16_- and C_24:1_-ceramides, especially when compared with the effect observed in response to C_6_-ceramide stimulation. To further characterize the molecular mechanism of apoptosis induction, we first analyzed changes in total cellular ceramide content, then endogenous C_16_- and C_24:1_-ceramide concentrations in response to known stimulatory agents such as mmLDL and *TNF-alpha* as references (Figure 1).^12^ We found that both, mmLDL and *TNF-alpha*, cause a significant rise in endogenous C_16_-ceramide (**2**), however, *TNFalpha* causes significant reduction of C_24:1_-ceramide concentrations in macrophages.

**Figure 2:**
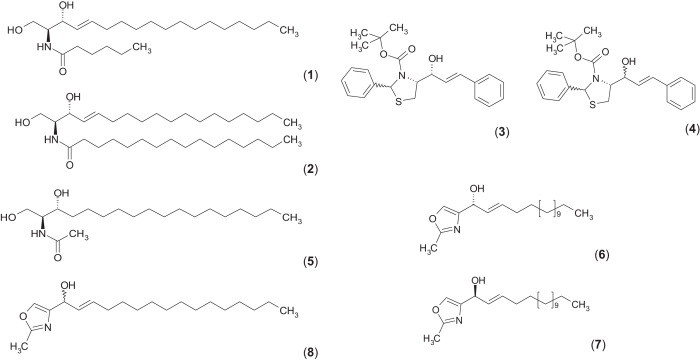
Natural ceramide and synthetic ceramide analogs. Chemical structures of C_6_-ceramide (**1**), C_16_-ceramide (**2**), C_2_-dihydroceramide (**5**) and synthetic ceramide analogs HPL-39N 4-[(1R)-(E)-1-Hydroxy-3-phenyl-allyl]-(2RS,4R)-2-phenyl-thiazolidin-3-carbonic acid-*t*-butylester (**3**), HPL-38N 4-[(1RS)-(E)-1-Hydroxy-3-phenyl-allyl]-(2RS,4R)-2-phenyl-thiazolidin-3-carbonic acid-*t*-butyl-ester (**4**), HPL-1R36N (1R)-(E)-(2-Methyl-oxazol-4-yl)-hexadec-2-en-1-ol (**6**) and HPL-1S36N (1S)-(E)-(2-Methyl-oxazol-4-yl)-hexadec-2-en-1-ol (**7**), HPL-1RS36N (1RS)-(E)-(2-Methyl-oxazol-4-yl)-hexadec-2-en-1-ol (**8**).

**Figure 3:**
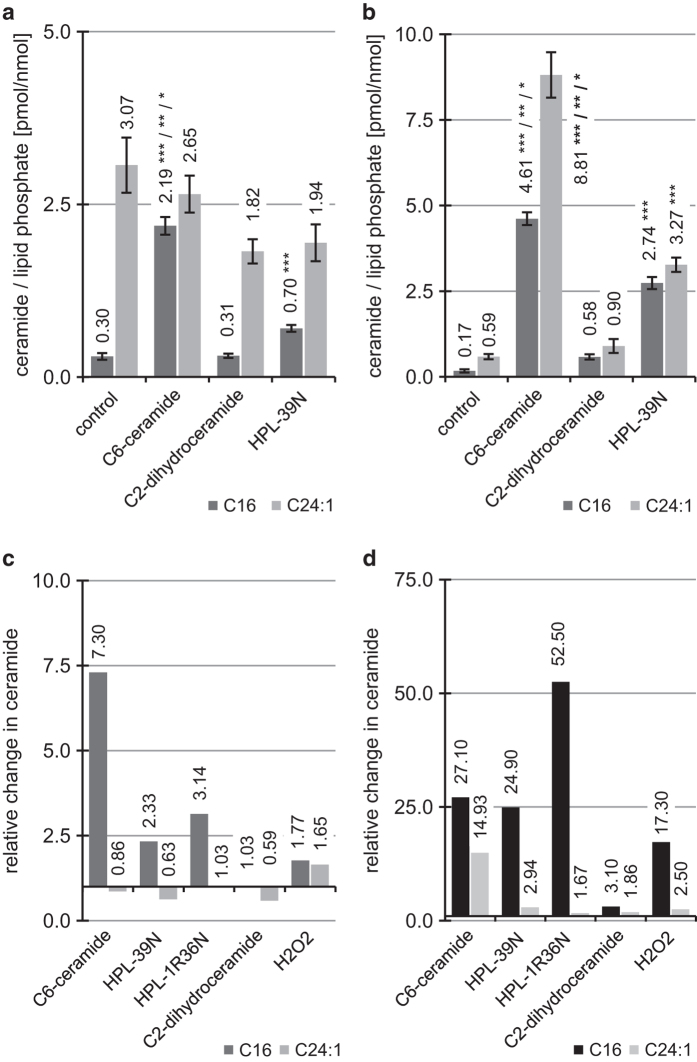
Induction and quantification of selected individual ceramide species in human macrophages and relative C_16_- and C_24:1_-ceramide concentrations after stimulation with compounds indicated. (**a**) Stimulation of C_16_- and (**b**) C_24:1_-ceramide concentration in macrophages after 4 and 7 h treatment with 10 *μ*M exogenous C_6_-ceramide (**1**), 10 *μ*M HPL-39N (**3**), 10 *μ*M C_2_-dihydroceramide (**4**), shown as mean±S.E., *n*=3. One-way ANOVA in combination with *post hoc t*-tests and Bonferroni's correction for multiple testing, *P*<0.0001 (one-way ANOVA); significant with *P*<0.05: *** *versus* control, ** *versus* C_2_-dihydroceramide (**4**), * *versus* HPL-39N (**3**). (**c**) Relative ceramide concentrations after 4 h and (**d**) 7 h stimulation. Relative ceramide concentrations are calculated based on data derived from **a** and **b**, and Figures 4c and d. H_2_O_2_ data obtained in experiments resulting in **a** and **b**. Values result from the ratios obtained from the ceramide content in response to the test agent *versus* ceramide content of untreated control (same incubation conditions, as indicated in Figures 3a and b, and Figures 4c and d. C_6_-ceramide data derived from 8 h incubation.

HPL-39N (4-[(1R)-(E)-1-Hydroxy-3-phenyl-allyl]-(2RS,4R)-2-phenyl-thiazolidin-3-carbon-säure-*t*-butylester) (**3**) (Figure 2) is an example of our novel series of bioactive conformationally stabilized ceramide analogs displaying superior pro-apoptotic and *PKC*-activating effects (see ref. 13 data not shown). The biologically almost inactive agent dihydroceramide was used as a control and known for hardly affecting apoptosis.^14^ Time-points for measurements were selected on the basis of published data obtained from Jurkat cells and U937 cells compatible with a time-delayed increase of C_16_-ceramide (**2**) in stimulated cells (with exogenous, cell permeable C_6_-ceramide (**1**)).^15,16^ Hence exogenous C_6_-ceramide (**1**) was used as a reference compound for synthetic, conformationally restricted ceramide analogs. HPL-39N (**3**) like C_6_-ceramide increases the total ceramide content (*P*<0.05, 8 h) from 0.76 pmol to 6.01 pmol (all values calculated as pmol ceramide/nmol lipid phosphate). Under these conditions applying a (under cell culture conditions) soluble control C_2_-dihydroceramide (**5**), fails to induce any significant effect on the total lipid content in macrophages (Figures 3a and b). In response to C_6_-ceramide, the C_24:1_-ceramide concentration is changed most significantly among ceramide species (Figure 3b).

### Apoptosis along with increased C_16_-ceramide concentrations

The three compounds tested, C_2_-dihydroceramide (**5**), exogenous C_6_-ceramide (**1**) and HPL-39N (**3**), not only differ in their apoptosis-inducing properties but also in their ability to change intracellular C_16_-ceramide concentration as determined by analyzing the lipid extract of macrophages. While exogenous C_6_-ceramide (**1**) and HPL-39N (**3**) induce a clear, statistically significant (*P*<0.05) increase in endogenous C_16_-ceramide concentration after 4 h, C_2_-dihydroceramide (**5**)-treated cells display no change (Figure 3a). Similarly, after 8 h (Figure 3b), a significant rise in the C_16_-ceramide concentration is noted, however, insignificant as for the C_2_-dihydroceramide (**5**)-induced effect (*P*<0.05). Compared with C_6_-ceramide, the effect of the racemic analogue HPL-39N (**3**) is significant as well but somewhat less pronounced (after 8 h: 2.19 pmol of C_16_-ceramide by C_6_-ceramide *versus* 2.74 pmol in response to HPL-39N (**3**)). Both the synthetic ceramide analogue HPL-39N (**3**) and its partial synthetic congener C_6_-ceramide (**1**) increase the C_24:1_-ceramide content in the cells after 8 h, whereas no significant change is observed after 4 h. This suggests that HPL-39 N (**3**) actually functions as a ceramide mimic and stimulates similar or identical mechanisms in the cell while C_2_-dihydroceramide (**5**) causes a drop in the C_24:1_-ceramide content (to 1.82 pmol C_24:1_-ceramide), it does not show a significant (*P*<0.05) increase in the C_24:1_-ceramide content after 8 h. Obviously the 4,5-trans double bond in the sphingosine backbone is an essential element for raising the cellular C_24:1_-ceramide content.

### Enantioselective effects of synthetic ceramide analogs HPL-1R36N (6) and HPL-1S36N (7) on endogenous ceramide species, caspase activation and apoptosis induction

The ceramide analogue HPL-39 N (**3**) bears an asymmetrically substituted hydroxy group while, unlike ceramides, the amino group is integrated into an oxazole heterocycle. To probe for stereospecific effects in terms of apoptosis induction, as well as on distinct endogenous ceramide species concentrations, compounds HPL-38 N (**4**) and HPL-1RS36N (**8**) were tested as racemates/diastereomeric mixtures, as well as enantiomers (obtained by separation on chiral columns (for HPL-1R36N (**6**)/HPL-1S36N (**7**)), alternatively by enantioselective synthesis (HPL-38 N (**4**), see Supplementary Materials). In contrast to the 1RS-racemate HPL-38 N (**4**), HPL-39 N (**3**) shows a strong apoptosis-inducing activity. At equal concentrations, the pure 1R compound HPL-39 N (**3**) is significantly more active than the racemic mixture with regard to apoptosis induction.^13^ The 1R compound HPL-39 N (**3**) (in comparison to exogenous C_6_-ceramide (**1**)) has significantly stronger effects on C_16_-ceramide concentrations as determined by analyzing the lipid extract of macrophages (Figure 3); both HPL-39 N (**3**) and its natural reference C_6_-ceramide (**1**) cause a significant increase in C_16_-ceramide content of macrophages and in parallel corresponding rates of apoptosis.

**Figure 4:**
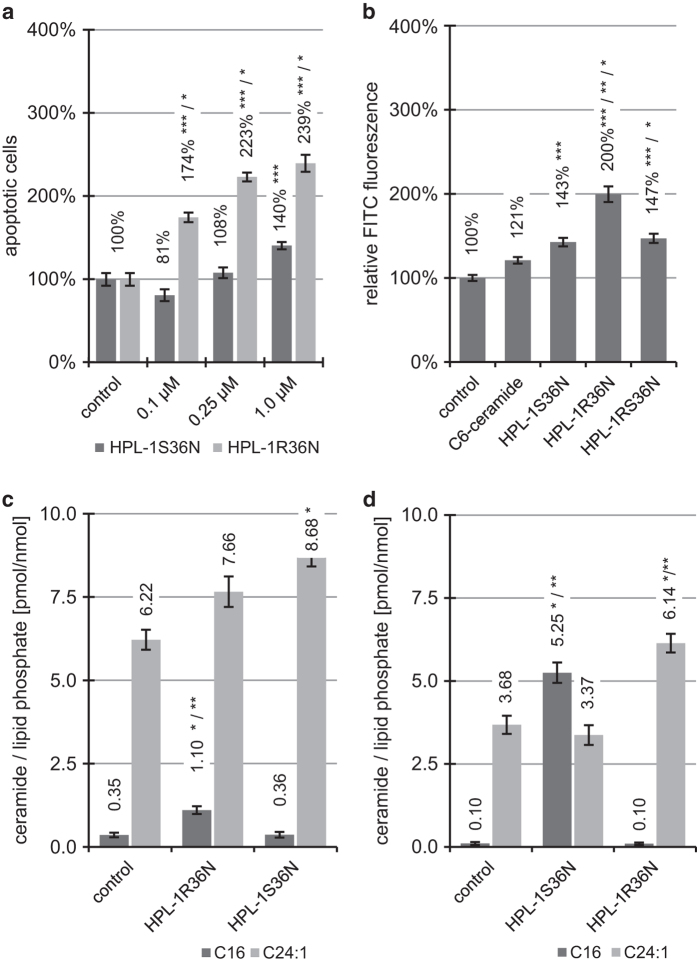
Diverging induction of apoptosis, caspase activity and ceramide concentrations of synthetic ceramide analogs in macrophages. (**a**) Macrophages were treated 4 h with 0.1, 0.25 and 1.0 *μ*M HPL-1R36N (**6**) or HPL-1S36N (**7**) and stained using YO-PRO-1 iodide (apoptotic cells) and Hoechst 33342 (complete cells). (**b**) Caspase activity (*in situ*-activated caspases labeled with *Casp*ACE-FITC-VAD-FMK) in isolated monocytes treated 16 h with 10 *μ*M HPL-1RS36N (**8**), HPL-1R36N (**6**), HPL-1S36N (**7**) and exogenous C_6_-ceramide (**1**) as positive control. Data shown as mean±S.E., *n*=3. One-way ANOVA in combination with *post hoc t*-tests and Bonferroni's correction for multiple testing, *P*<0.0001 (one-way ANOVA); significantly different with *P*<0.05: *** *versus* control (**a** and **b**), ** *versus* HPL-1RS36N (**8**) (**a**),* *versus* HPL-1S36N (**7**) (**a**) or exogenous C_6_-ceramide (**1**) (**b**). (**c**) Macrophages were treated 4 h and (**d**) 7 h with 10 *μ*M HPL-1R36N (**6**) and HPL-1S36N (**7**), C_16_- and C_24:1_-ceramide concentrations shown as mean±S.E., *n*=3. One-way ANOVA in combination with *post hoc t*-tests and Bonferroni's correction for multiple testing, *P*<0.0001 (one-way ANOVA); significant with *P*<0.05: * *versus* control, ** *versus* HPL-1S36N (**7**).

To further examine the effect of the absolute configuration of the asymmetrically substituted C1 carbon on biological activity, the enantiomers of HPL-1RS36N (**8**), mimicking the sterically stabilized sphingosine backbone of natural ceramides, were analyzed in more detail. Racemic HPL-1RS36N (**8**), was separated into the 1R- (**6**) and the 1S- enantiomer (**7**) by use of a chiral cyclodextrin column (mobile phase: ethyl acetate/n-hexane) to afford HPL-1S36N (1*S*)-(E)-(2-methyl-oxazol-4-yl)-hexadec-2-en-1-ol (**6**) and HPL-1R36N (1R)-(E)-(2-methyl-oxazol-4-yl)-hexadec-2-ene-1-ol (**7**). In line with their apoptosis-inducing potency, the synthetic ceramide analogs activate caspases more strongly than exogenous C_6_-ceramide (**1**) (Figure 4b), possibly due to increased metabolic stability and increased half-life of the synthetic compound. Similar to HPL-39 N (**3**), the racemate HPL-1RS36N (**8**) exhibits a stronger pro-apoptotic activity, as well as a PKCalpha-activating effect when compared with exogenous C_6_-ceramide (**1**).^13^

### Apoptosis induction reveals stereochemistry-dependent effects (analysis of HPL-38 N (1RS) (**8**)- and HPL-39 N (1R) (**3**)-induced effects)

Similar to HPL-39 N (**3**), the two enantiomers of HPL-1RS36N (**8**) affect apoptosis in human macrophages to different degrees. At all tested concentrations, the effect of the 1R enantiomer HPL-1R36N (**6**) on apoptosis was significantly stronger (*P*<0.05) when compared with HPL-1S36N (**7**) (Figure 4a). The R-enantiomer HPL-1R36N (**6**) induces apoptosis to a greater extent (1.4 times stronger, *P*<0.05) when compared with the S-enantiomer HPL-1R36N (**6**). HPL-1S36N (**7**) exhibited a statistically significant (*P*<0.05) effect only at the maximum concentration used (1.0 *μ*M). Concentration dependency is only statistically significant for HPL-1R36N (**6**) (*P*<0.05). When comparing activation of caspases (Figure 4b) as reflected by FITC-fluorescence, a similar picture emerges with a significantly greater effect associated with administration of the R-enantiomer. Caspase-independent pathways await further examination.

### Stereochemistry at C1 has profound impact on the formation of endogenous C_16_-ceramide

About 10 *μ*M of the pure enantiomers HPL-1R36N (**6**) and HPL-1S36N (**7**) were tested (4 and 7 h) on macrophages to determine their effects on endogenous C_16_-ceramide (**2**), as well as C_24:1_-ceramide concentration. In fact, the stereochemistry at C1 exhibits a profound impact on the formation of C_16_-ceramide (**2**). HPL-1R36N (**6**), the 1R-enantiomer of HPL-1RS36N (**8**), increases the C_16_-ceramide concentration in the lipid extract of the cells after 4 h (*P*<0.05, from 0.35 pmol C_16_-ceramide (control) to 1.10 pmol of C_16_-ceramide), four times the value of the untreated control (Figure 4c). The 1S-enantiomer HPL-1S36N (**7**), however, is almost inactive under these conditions. After 7 h, the difference between both enantiomers is even more pronounced (Figure 4d). Only HPL-1R36N (**6**) exhibits a strong (*P*<0.05) increase in C_16_-ceramide concentration to 5.25 pmol/nmol lipid phosphate, to >50 times the initial value.

HPL-39 N (**3**) and HPL-1R36N (**6**) are both 1R-enantiomers at the C1 carbon; at equal concentrations (7 h), however, the effect of the latter is significantly more pronounced (5.25 pmol C_16_-ceramide HPL-1R36N (**6**) *versus* 2.74 pmol C_16_-ceramide (HPL-39 N (**3**)). Both agents differ in the functional groups adjacent to the exocyclic trans-2,3-double bond (corresponding to the 4,5-trans double bond in the sphingosine backbone of natural ceramide). The aliphatic, unsubstituted dodecanoyl agent proved more efficient than the phenyl-substituted congener in terms of C_16_-ceramide (**2**) induction. Notably, in contrast to C_16_-ceramide (**2**), the two enantiomers HPL-1R36N (**6**) and HPL-1S36N (**7**) differed in their effects on the C_24:1_-ceramide concentration over the observation period (Figure 4): no significant effect was noted with HPL-1S36N (**7**) after 4 h, whereas with HPL-1R36 (**6**) the C_24:1_-ceramide concentration increased slightly (*P*<0.05) to 8.68 pmol C_24:1_-ceramide. After 7 h, the C_24:1_-ceramide concentration was 6.14 pmol, well above the level of the control (3.68 pmol). Thus, unlike C_16_-ceramide, a 1S stereochemistry-dependent increase with regard to C_24:1_-ceramide is evident. When the strength of the observed biological effect (change of C_16_- and C_24:1_-ceramide concentrations), is compared with the reference exogenous C_6_-ceramide (**1**) at equal concentrations (Figures 3c and d), then HPL-39 N (**3**) and HPL-1R36N (**6**) can be classified as less effective at 4-h incubation time.

**Figure 5:**
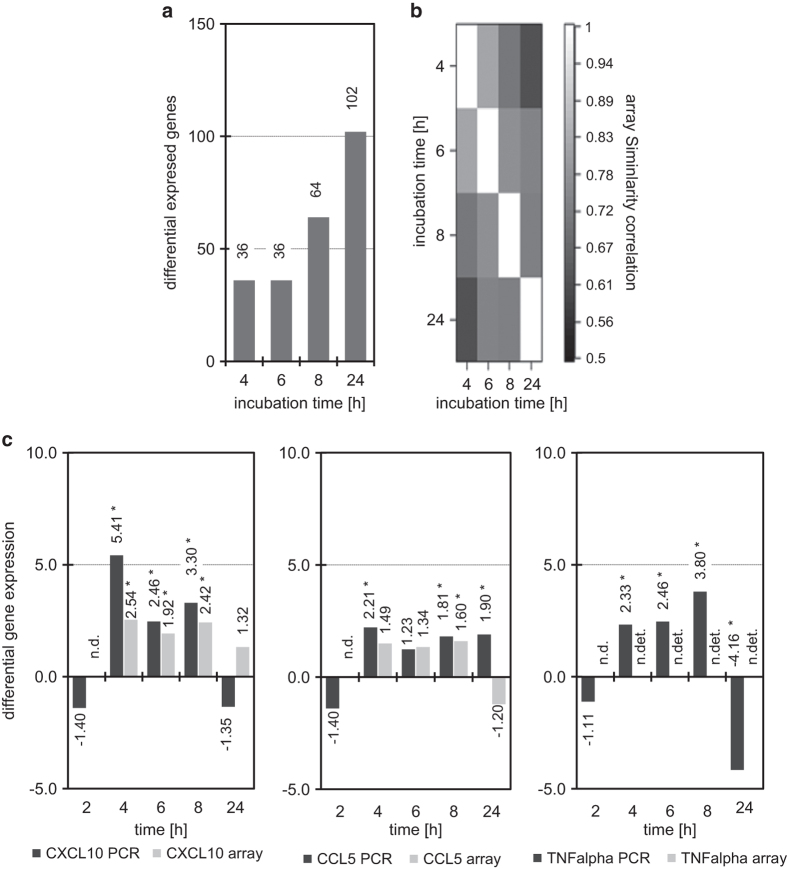
Synthetic ceramide analogs affect differential gene expression in HT-29 cells. HT-29 cells were treated 2, 4, 6, 8 and 24 h with 30 *μ*M HPL-1R36N (**6**) and HPL-1S36N (**7**). (**a**) Differential expressed genes in total RNA of HT-29 cells. Total RNA of cells treated 4, 6, 8, 24 h with HPL-1R36N (**6**) and HPL-1S36N (**7**) were transcribed and labeled separately, however, co-hybridized on the same array. (**b**) Array similarity matrix of hybridized oligonucleotide arrays. Therefore, array dissimilarity in similarity matrix (Pearson’s sample correlation) shows similar behavior as differential gene expression caused by the individual enantiomers. (**c**) *CXCL10*, *CCL5* and *TNF-alpha*: three prominent differentially expressed genes in real-time PCR (2, 4, 6, 8, 24 h) and spotted oligonucleotide microarray (4, 6, 8, 24 h) on treatment of HT-29 cells with synthetic ceramides. Significance is supposed, if numerical value of differential gene expression >1.5 fold. Not determined (n.d.), not detectable (n.det.) due to low specific mRNA concentration.

However, in contrast, the effect of HPL-1R36N (**6**) is quite strong when pro-apoptotic activity is considered. HPL-1R36N (**6**) significantly induces apoptosis (*P*<0.05) (Figure 4a). Over a maximum incubation period of 7 h (HPL-39N (**3**) and HPL-1R36N (**6**)) resp. 8 h (C_6_-ceramide (**1**)), HPL-39 N (**3**) is approximately as efficient as C_6_-ceramide (**1**), and HPL-1R36N (**6**) is approximately twice as potent as exogenous C_6_-ceramide (**1**). This observation may be explained by responses in terms of total amounts of pro-apoptotic C_16_-ceramide generated (amount determined as the integral of the time–concentration curve), causing an extended activation of caspases by HPL-1R36 (**6**) (Figure 4b) and induction of apoptosis-related genes in comparison to C_6_-ceramide (**1**) (Figure 5c).

### Signaling mechanisms behind enantioselective apoptosis induction

Since apoptosis induction is of outstanding relevance to mechanisms of cancer treatment, we further examined agent-associated enantiospecific effects on the transcriptional level. Human HT-29-cells (Caucasian colon adenocarcinoma grade II), a widely used cancer model, were treated with HPL-1R36N (**6**) and HPL-1S36 (**7**) and effects analyzed by spotted microarrays (Lab Arraytor 60-1 to 60-6 combi oligonucleotide chip (SIRS-Lab, Jena, Germany), Figures 5a and b).^17^ Interestingly the enantiomers 1(R)-(E)-(2-methyl-oxazol-4-yl)-hexadec-2-en-1-ol (**6**) and 1(S)-(E)-(2-methyl-oxazol-4-yl)-hexadec-2-en-1-ol (**7**) of the bioactive oxazol compound HPL-1RS36N (**8**), differed markedly as for time- and enantiomer-specific effects on transcription. On array analysis, 158 genes showed RNA-concentration changes ≥factor 1.5 and were assigned as differentially regulated; 16 gene activities showing more than 2-fold changes were identified. Microarray analysis thus revealed that differences in molecular mechanisms of enantiomers can be directly detected at the transcriptional level. Notably key players of apoptotic pathways are affected differently and apparently reflect pro-apoptotic potency. As for synthetic ceramide analogs, the data indicate that tiny structural changes may be crucial and transcriptomics appears valuable for compound characterization and optimization of efficacy in this context.

### Enantioselectively inducible genes—stereospecificity of ceramide analogs

Similar to HPL-39 N (**3**), HPL-1RS36N (**8**) also activates PKCalpha more strongly than C_6_-ceramide (**1**), and exhibits a larger pro-apoptotic activity in Renca cells.^13^ Human colon carcinoma cells HT-29 are a well-established model to study ceramide-triggered effects on proliferation and induction of apoptosis.^17^ In human HT-29 cells, the two enantiomers of HPL-1RS36N (**8**)—HPL-1R36N (**6**) and HPL-1S36N (**7**)—show distinct effects on gene expression. HT-29 cells, however, are adherent human colon adenocarcinoma cells, much less metabolically active compared with mononuclear leukocytes or monocytes. So it is hardly surprising that, ultimately, only a few differentially expressed genes were identified among the totally 593 human genes spotted on the microarray. Out of a total of 28 genes, 12 were found to be differentially expressed as analyzed by quantitative real-time PCR at one or more points in time: as for their biological function these can be classified into three groups: (I) transcription factors affecting cell cycle regulation (*E2F1*, *E2F2*, *E2F5*, *E2F6*), (II) enzymes and proteins involved in apoptosis (*ASAH1*, *ITPK1*, *BCL2L11*, *TRAF4*) and (III) chemo- and cytokines (*CCL5*, *CXCL10*, *IL-1B*, *TNF-alpha*), known to be involved in cancer development, cell death, cell growth, cellular development and cell cycle.

## Discussion

In light of the directly opposed effects of FKBP51 and FKBP52 inhibitors,^18^ selective and metabolically stable synthetic probes are critical for mechanistic studies, as well as for assessing the pharmacological potential of ceramide mimics. Apart from corresponding structural functionalities, the synthetic ceramide analog HPL-1RS36N (**8**) shares similar alteration of target transcripts with ceramide (MB, MK and H-PD, unpublished experiments)). Since the R-enantiomer HPL-1R36N (**6**) is significantly more effective, the stereochemistry at the C1-atom, in addition to the 2,3-trans-double bond, proves to be an essential structural feature. We demonstrate that under our experimental setting, three genes showing differential regulation across all time points were identified by both hybridization experiments (*CXCL10* and *CCL5*) and quantitative real-time PCR (*TNF-alpha*). All of these differentially regulated genes are known for their critical role in apoptosis and cell cycle control. *CXCL10* belongs to the group of chemokines without ELR motif, and is part of the immune and lymphatic system and the immune response; it has signal transducing, receptor binding, chemokine and chemo-attractant activity and is only present extracellularly. *CXCL10* is further involved in the cell cycle, inter-cellular communication, in cell growth, cell proliferation and movement, angiogenesis (preventing angiogenesis), apoptosis (detected in neurons) and carcinomas.^19^ Our agents and structurally related compounds thus may target cells capable of producing *CXCL10* such as adenocarcinoma cells. In human intestinal epithelial cells *IFN-gamma* induces *NF-κB*, *IL1* and *TNF-alpha* expression and secretion of *CXCL10*. *CXCL10* itself, however, exhibits no direct effect on HT-29 cells since *CXCL10* is not a ligand of the (only proven) *CXCR4* receptor. The associated *CXCL10* receptor *CXCR3* appears to be as little expressed in HT-29 cells as the specific ligand for *CXCR4*, the *CXCL12* receptor.^20^ *CXCL10*, however, has been shown to induce caspase-3-dependent apoptosis in neurons, which is blocked irreversibly by the caspase-3 inhibitor DEVD and VAD.^21^ If VAD is coupled with a fluorescent dye (e.g. CaspACE-FITC-VAD-FMK), activated caspases can be measured by flow cytometry.^22^ In a flow cytometry experiment in *CXCR3* receptor-positive cells, the two enantiomers HPL-1R36N (**6**) and HPL-1S36N (**7**) (Figure 4b) should accordingly exhibit differences in terms of activated caspases. A representative result for human macrophages (*CXCR3* receptor positive) is shown in Figure 4b. Similar to *CXCL10* expression, HPL-1R36N (**6**) is the more active enantiomer. When comparing differences in *CXCL10* expression, caspase activation and apoptosis induction, the differences in caspase activation are relatively moderate (~1.4-fold). Accordingly, it is reasonable to assume that apoptosis-inducing effects in this context not only rely on the activation of caspases, but also on caspase-independent signaling, subject to future experimentation.

*CCL5* (RANTES) is another cytokine found to be differentially regulated by the two enantiomeric ceramide analogs (Figure 5c). It acts as an immunoregulant, chemotactic agent (inducing migration) by attracting T lymphocytes (T cells) of the Th1- and memory cell type on eosinophilic and basophilic granulocytes. It thus controls the recruitment of leukocytes into inflamed tissue. Apoptosis induction in peripheral blood T cells via *CCR5* receptor occupation can be achieved with *CCL5* concentrations in the micromolar range; signal transduction here is initiated by cytochrome *c* release via caspase-3- and caspase-9- and *PARP-1*-dependent pathways.^23^ However, in the absence of the *CCR5* receptor—as in HT-29 cells—apoptosis cannot be induced. When interpreting the results of the flow cytometric experiments with the enantiomers (Figure 4b) in human macrophages it has to be considered that although these cells belong to the *CCR5* receptor-positive ones, the pro-apoptotic effect of *CCL5* is negligible due to the necessarily very high *CCL5* concentration.

*TNF-alpha* (*TNFSF2*) is the third prominently altered cytokine transcript. In contrast to *CXCL10* and *CCL5*, differential *TNF-alpha* expression, due to low concentration of its mRNA, has been detectable only by quantitative real-time PCR. TNF-alpha is a multifunctional pro-inflammatory cytokine belonging to the TNF super family playing a central role in a variety of biological processes such as apoptosis, induction of the expression of genes, lipid metabolism, secretion of cytokines, cell activation, cell death, cell adhesion, cell differentiation, cell stimulation and cell proliferation.^24^

As a common feature, biological effects mediated by the three genes *CXCL10*, *CCL5* and *TNF-alpha* are exclusively conveyed through binding to specific receptors. HT-29 cells express only *TNFR1* and *TNFR2* cytokine receptors for *TNF-alpha* and are therefore irresponsive to biological effects of *CXCL10* and *CCL5* including apoptosis induction. However, many cells in their natural tissue environment express these cytokine-specific receptors, and apoptosis can be initiated in response to secretion by, for example, adenocarcinoma cells. We thus have been able to identify several potential contributors to observed alterations in apoptosis along with raised concentration of C_16_-ceramide and have shown that caspase activation may be a critical component. An analysis of further details and (quantitative) degree of distinct contributions were beyond the scope of this report, however, will be addressed by future investigations.

Our data demonstrate that enantiospecific ceramide-like signaling and apoptosis of tumor cells can be induced by administration of synthetic probes with defined stereochemistry specifically raising the level of apoptogenic endogenous C_16_-ceramide. Enantioselective effects on transcription induced via these agents also demonstrate their utility as specific probes for elucidating molecular mechanisms of apoptosis signaled via distinct endogenous ceramide species. Further, this study is likely to stimulate further research toward structurally related anti-tumor agents, for example, capable of inducing *CXCL10* in tumor cells (**1**).

## Materials and Methods

### Cell culture and cell culture experiments

#### Monocytes/macrophages

HMBC used in cell culture experiment were isolated from fresh buffy coats using Histopaque (Sigma-Aldrich, Deisenhofen, Germany) and cultured in six-well plates (9.6 cm^2^, Greiner Bio-One, Frickenhausen, Germany) six to seven days in a humidified incubator at 37 °C and 5% CO_2_ with RPMI-1640 medium (PAA, Cölbe, Germany) supplemented with mit 2 mM L-glutamine, 50 U/ml penicillin 50 *μ*g/ml streptomycin (Sigma-Aldrich) and 10% FCS (Greiner Bio-One). Before and during incubation with compounds supplemented with RPMI-1640 Medium with a reduced content of 1% of FCS was used. Human colon carcinoma cells HT-29 (DSMZ, Braunschweig, Germany) were cultured in cell culture flasks (175 or 25 cm^2^ (stock) in a humidified incubator at 37 °C and 5% CO_2_ with McCoy´s 5 A Medium (PromoCell, Heidelberg, Germany) supplemented with mit 2 mM L-glutamine, 50 U/ml penicillin, 50 *μ*g/ml streptomycin and 10% FCS. Before experiments cells were seeded semi-confluent in 5.8 cm^2^ Petri dishes (Greiner Bio-One), equilibrated at 37 °C over night in McCoy´s 5 A Medium with 1% human AB serum and supplemented with 2 mM L-Glutamine, 50 U/ml Penicillin and 50 *μ*g/ml Streptomycin.

#### Ceramide quantification

Macrophages were treated 4 h with mmLDL (54 *μ*g/ml) and TNF-alpha (3 ng/ml) (Figure 1), 4 or 8 h with 10 *μ*M exogene C_6_-ceramide (**1**), 10 *μ*M HPL-39 N (**3**), 10 *μ*M C_2_-dihydroceramide (**5**) (Figure 3) or treated 4 or 7 h with 10 *μ*M HPL-1R36N (**6**) and HPL-1S36N (**7**) (Figure 4). All organic compounds were dissolved in a 10 mM ethanol/DMSO stock solution; the same concentrations of solvents were present in control samples. Appropriate dilutions in ethanol/cell culture medium and volumes were applied to the cells at the beginning of incubation. Lipid extraction, purification and ceramide quantification were performed by HPLC-analysis as described in section ceramide quantification using DECCA (7-diethylamino)coumarin-3-carbonyl azide) for ceramide labeling and 100 pmol C_8_-ceramide as internal standard.^25^

#### Oligonucleotide array/quantitative real-time PCR

HT-29 cells were incubated 4, 6, 8 and 24 h (oligonucleotide array) or 2, 4, 6, 8 and 24 h (real-time PCR) with 30 *μ*M HPL-1R36N (**6**) and HPL-1S36N (**7**).

#### Apoptosis

Macrophages were cultivated in RPMI-1640 without phenol red (Invitrogen, Karlsruhe, Germany) and treated 4 h with 0.1, 0.25 and 1.0 *μ*M HPL-1R36N (**6**) or HPL-1S36N (**7**). YO-PRO-1 iodide (491/509) (0.1 mM in DMSO/PBS-buffer, staining apoptotic cells) and Hoechst 33342 (0.1 mM in DMSO/PBS-buffer, staining complete cells) (Molecular Probes, Leiden, Netherlands or MobiTec, Göttingen, Germany) for apoptosis/number of cells was applied to medium at the end of incubation time. Percentage of apoptotic cells was determined on a Nikon Eclipse FS 100 equipped with an EPI-filter block 340–380 nm or 450–490 nm (Nikon Instruments Europe, Düsseldorf, Germany).

#### PARP fragmentation/CaspACE FITC-VAD-FMK *in situ* marker labeling

To confirm apoptosis in ongoing in cells by PARP-fragmentation analysis applying densitometric quantification of fragment formation, isolated monocytes were treated 16 h in supplemented RPMI-1640 medium with 10 *μ*M HPL-1RS36N (**8**), HPL-1R36N (**6**), HPL-1S36N (**7**) and exogenous C_6_-ceramide (**1**) as positive control. CaspACE FITC-VAD-FMK *In Situ* Marker (Promega, Mannheim, Germany) was added directly to the cell culture medium at a final concentration of 5 *μ*M and incubated 20 min at 20 °C. Washing, fixing and flow cytometry analysis on a FACScan (Becton Dickinson, Heidelberg, Germany) were performed according manufacturer's instructions. Fluorescence was measured at an emission of 530 nm (excitation of 488 nm).

### Ceramide quantification

Reagents and chemicals: C_6_-, C_8_-, C_16_-, C_18_-, C_24_-ceramide, C_2_-dihydroceramide and SM were obtained from Sigma-Aldrich, Alexis (Grünberg, Germany) or Acros (Geel, Belgium), and DECCA and water-free toluene (over molecular sieve 4 A°) were obtained from Fluka (Taufkirchen, Germany). Methanol, ethyl acetate, cyclohexane, acetonitrile and iodine were supplied from Merck (Darmstadt, Germany), chloroform by J.T. Baker (Deventer, Netherlands). All chemicals were of reagent grade. Solvents for HPLC were either freshly distilled or commercially available HPLC grade.

#### Lipid extraction

Lipid extraction was performed according to the method of Bligh and Dyer.^26^ After incubation, cells were isolated and washed twice with ice-cold PBS, 500 *μ*l ice-cold PBS added, cells scraped off the plates (after addition of PBS), transferred to 1.5 ml tubes (Eppendorf, Hamburg, Germany), centrifuged, the PBS supernatant carefully removed, re-suspended in 150 *μ*l methanol and cellular lipids extracted or the pellet stored at −80 °C. The resulting crude lipid extract is split in 50 *μ*l for lipid phosphate quantification and 100 *μ*l for ceramide analysis. Phosphate quantification is performed according to the book by Jenkins and Hannun.^27^ To separate ceramides from other (sphingo-)lipids, the crude extract is separated on a 0.2 mm silica gel 60 F254-coated TLC plate (Merck) using a 6 : 4 : 1 (v/v) chloroform/methanol/water mixture as mobile phase. Exogenous ceramides are used as TLC plate marker, lanes separated from cellular lipids and detected separately in an iodine chamber. Ceramide bands (cellular lipids) are scraped from the plate and transferred to Poly-Prep Columns (Bio-Rad, Munich, Germany), eluted with chloroform (3×250 *μ*l) and finally with 250 *μ*l methanol. Glycerolipid/monoacylglycerol impurities are removed by alkaline hydrolysis in 0.03 M NaOH in 90% ethanol at 37 °C for 30 min. After neutralization a second Bligh and Dyer lipid extraction is performed. The organic phase is dried over water-free sodium sulfate and evaporated to dryness.

#### Ceramide labeling and HPLC separation

An optimized HPLC method based on conditions similar to those by Moersel and Balestrieri was used to achieve best performance in terms of sensitivity and ceramide species separation.^25,28,29^ Solutions containing 100 pmol reference C_8_-ceramide (for calibration) and ceramides obtained from cellular lipid extracts (equivalent to 5 to 10 nmol lipid phosphate or (5, 10, 25, 50, 100, 250 and 500 pmol of C_16_-ceramide) are transferred to sealed borosilicate glass vials with 100 *μ*l inset (VWR, Darmstadt, Germany), solvents evaporated (SpeedVac Concentrator, Eppendorf), the residue re-suspended in 10 *μ*l 0.3 mg/ml DECCA in toluene and heated (6 h, 80 °C) under shaking. After cooling to room temperature and solvent evaporation (SpeedVac Concentrator), the orange-red residue is dissolved in 50 *μ*l acetonitrile HPLC grade and subjected to HPLC separation. Labeled cellular ceramides are separated on a LiChrospher 100 (5 *μ*m) 250-4 RP-18 HPLC-Column (Merck) with 10 *μ*l injection volume (autosampler Spark Promis II (Spark, Friedrichsdorf, Germany) with a gradient solvent system: (acetonitrile-methanol-ethyl acetate composition (v/v)): 0.0–10.0 min: isocratic 50:45:5; 10.0–15.0 min to 45:50:5; 15.0–20.0 min to 40:50:10; 20.0–60.0 min to 30:50:20; 60.0–65.0 to return to starting conditions; 65.0–80.0 equilibration at starting conditions prior to next injection (flow rate: 1 ml/min, Merck Hitachi HPLC-pump L6200 A (Merck), fluorescence detection with a Shimadzu RF–535 fluorescence detector (Shimadzu, Duisburg, Germany) at an experimentally determined excitation wavelength of 380 nm and emission wavelength of 475 nm).

### Total RNA sample preparation

Monolayers were washed with ice-cold PBS and cells were scraped in 3 ml lysis buffer and total RNA from HT-29 cells was extracted with RNeasy Mini Spin Columns (Qiagen, Hilden, Germany) or TriPure Isolation Reagent (Roche Diagnostics, Mannheim, Germany) according to the manufacturer's instructions. RNA yields were determined spectrophotometrically on a NanoDrop ND-1000 Spectro-Photometer (NanoDrop Technologies, Wilmington, USA) by measuring the absorbance at 260 and 280 nm. All RNA samples used for microarray analysis were analyzed by ethidium bromide-stained RNA agarose gel, to confirm purity and integrity of RNA, and a Bioanalyzer 2100 (Agilent Technologies, Boeblingen, Germany).

### Oligonucleotide microarray hybridization

Experiments were performed using the Lab-Arraytor-60-1 to 60-6 combi oligonucleotide microarray (SIRS-Lab), comprising 539 probes (each made as triple replicates) addressing 519 transcripts corresponding to inflammation, as well as 22 reliable control probes (see Supplementary Notes: ‘Microarray Experiment Description’ online according to ref. ^30^ About 10 *μ*g of total RNA were reverse transcribed using Superscript-II reverse transcriptase from Invitrogen in the presence of aminoallyl-dUTP from Sigma (Taufkirchen, Germany) and labeled using the Dyomics DY-648-S-NHS/DY-548-S-NHS labeling system (Dyomics, Jena, Germany). DY-548-S-NHS-labeled cDNA from HPL-1S36N (**7**)-treated cells were co-hybridized with DY-648-S-NHS-labeled cDNA obtained from the same amount of total RNA isolated from cells treated with HPL-1R36N (**6**). After incubation in a hybridization apparatus (HS 400, TECAN, Crailsheim, Germany, for 10 h at 42 °C, formamide-based hybridization buffer system) arrays were washed according to the manufacturer’s instructions, dried and hybridization signal intensities were measured immediately using an Axon 4000B scanner (Axon Instruments, Foster City, CA, USA). Microarray data pre-processing of hybridization signals included (i) spot detection and background subtraction, (ii) spot flagging according to defined signal-to-noise threshold values and (iii) normalization and transformation of the signals obtained from different channels. For the former two steps, the GenePix 5.0 Analysis Software 5.0 (Axon Instruments) was used; for the third step we applied the approach from the study by Huber *et al*.^31,32^

### Quantitative real-time PCR

#### cDNA synthesis

First-strand complementary DNA synthesis was performed with 2 *μ*g of isolated total RNA from a HT-29 cells (also used for hybridization experiments) according to the manufacturer’s instructions. After adjusting total RNA volume to 11 *μ*l, 1 *μ*l (2.5 *μ*g/*μ*l oligo-d(T)12-18-primer solution is added, denatured at 70 °C in a PTC-200 DNA Engine (Bio-Rad) for 10 min and chilled on ice for 5 min. About 9 *μ*l of RT-Mix (4 *μ*l reaction-buffer 5×, 2 *μ*l DTT 100 mM, 0.5 *μ*l RiboLock RNAse-inhibitor 40 U/ml,1 *μ*l Revert Aid Reverse Transcriptase (RT)), 10 *μ*l 10 mM dNTP stock solution (10 mM dATP, 10 mM dGTP, 10 mM dCTP, 10 mM dTTP) (Thermo Scientific Molecular Biology, St. Leon-Rot, Germany) added, mixed and incubated 60 min at 42 °C. The enzyme was then inactivated by incubation at 70 °C for 5 min.

#### Quantitative real-time PCR

Real-Time PCR was performed in a Bio-Rad iQ-cycler (Bio-Rad) equipped with skirted Micro seal 96-Well PCR-plates covered with Microseal ‘B’ adhesive foils (Bio-Rad). Reaction volume (20 *μ*l, containing 30 ng cDNA) consists of 4 *μ*l diluted cDNA-solution (7.5 ng/*μ*l), 2 *μ*l 0.1 *μ*M gene-specific forward and reverse primer solution (2 pMol each; Biomers, Ulm, Germany), 4 *μ*l DEPC-treated water and 10 *μ*l RT^2^ Real-Time SYBR-Green/Fluorescein-PCR-Master-Mix (SA Bioscience, Hilden, Germany/Qiagen). According to manufacturer recommendations, a cycling program with an initial activation step (94 °C, 3 min) followed by 45 cycles of denaturation (94 °C, 30 s), annealing (60 °C, 30 s), elongation (72 °C, 30 s) and a final elongation (72 °C, 30 s) is executed. Fluorescence acquisitions in the SYBR green and ROX (internal reference dye) channels were performed at the end of the annealing step. A melting protocol ranged from 94 to 59 °C following a stepwise increment of 0.5 °C held for 3 s. Each sample as well as a negative template control (NTC) was amplified in triplet for each of the primer pairs assayed. Raw data (ct-values) were extracted using iCycler iQ-software (version 3.1, Bio-Rad) running on the Bio-Rad iQ-cycler.

### Statistics

Preprocessing and statistical analysis of microarray gene expression data were performed using the statistical software R in combination with Bioconductor.^33,34^ qBasePlus-Software (Version 1.3; Biogazelle, Gent, Belgium) was used for RT-qPCR data. Statistical significance was investigated using one-way ANOVA in combination with *post hoc t*-tests and Bonferroni's correction for multiple testing, *P*<0.0001 (one-way ANOVA); significant difference was considered if *P*<0.05. For more details we refer to the Supplementary Material.

### Primer design, synthesis, data read out and sequences

Gene specific primers (18–22-bp length) were designed by use of Primer 3 software version 0.4.0 (MIT Center for Genome Research; http://frodo.wi.mit.edu/cgi-bin/primer3/primer3_www.cgi) to obtain an annealing temperature of 57 °C and an amplicon length between 50 and 150 bp (up to 250 bp if necessary).^35^ Gene and species specificity was tested using NCBI nucleotide database, nucleotide blast and interrogation mode ‘blastn’ (the National Center for Biotechnology Information, Bethesda, MD, USA).

### Raw data extraction, normalization software/reference genes

Relative gene expression for each investigated gene was calculated using qBasePlus-Software (Version 1.3, Biogazelle) or the method of Pfaffl.^36^ Primer efficiency was determined for each primer by a cleaned up PCR-product dilution series (QIAquick PCR Purification Kit, Qiagen). HPRT1 was chosen as a reference gene for normalization of relative gene expression of each gene. It was selected as the most stable gene of all tested genes by ‘NormFinder’-algorithm.^37^ Stability value was calculated as 0.200±0.094.

#### Primer sequences

Each primer is characterized by the following characteristic features: symbol/gene/gene bank Accession/primer sequence forward (5ʹ to 3ʹ) (fw), reverse (5ʹ to 3ʹ) (rev)/amplicon size.

#### Genes-of-interest found differentially expressed

*ASAH1*: *N-Acylsphingosine amido-hydrolase (acid ceramidase) 1*; NM_177924.3 (transcript variant 1), NM_004315.4 (transcript variant 2); fw: 5ʹ-CCTCTGTACGTTGGTCCTGAA-3ʹ; rev: 5ʹ-GGCCTCCTACCCAAGTCTCA-3ʹ; 135 bp.

*BCL2L11*: *BCL2-like 11 (apoptosis facilitator)*; NM_207002.2 (transcript variant 9); fw: 5ʹ-CTACAGACAGAGCCACAAGA-3ʹ; rev: 5ʹ-ATCCAAAGCACAGTGAAAGA-3ʹ; 154 bp.

*CCL5*: *Chemokine (C-C motif) Ligand 5*; NM_002985.2; fw: 5ʹ-ATCCTCATTGCTACTGCCCTC-3ʹ; rev: 5ʹ-GCCACTGGTGTAGAAATACTCC-3ʹ; 135 bp.

*CXCL10*: *Chemokine (C-X-C motif) Ligand 10*; NM_001565.2; fw: 5ʹ-TGGATGTTCTGACCCTGCTTC-3ʹ; rev: 5ʹ-GGCAGTGGAAGTCCATGAAG-3ʹ; 175 bp. E2F1: E2F Transcription Factor; NM_005225.2; fw: 5ʹ-CTGCTCTTCGCCACACCGCA-3ʹ; rev: 5ʹ-TCCAGGTCCAGCCTCCGCTT-3ʹ; 95 bp.

E2F2: *E2F Transcription Factor* *2*; NM_004091.2; fw: 5ʹ-AAGTTGTGCGATGCCTGCCG-3ʹ; rev: 5ʹ-GCAGCCCCAGCGAAGTGTCATA-3ʹ; 192 bp.

E2F5: *E2F Transcription Factor 5,* *p130-binding*; NM_001951.3 (transcript variant 1), NM_001083588.1 (transcript variant 2); fw: 5ʹ-AGCAGGCACGAGAAGAGCCT-3ʹ; rev: 5ʹ-ACAGCCAAAGTATCAGCAGCCG-3ʹ; 110 bp.

*E2F6*: *E2F Transcription Factor 6*; NM_198256.2; fw: 5ʹ-TTTCCGTCTGCGTCGGGAGC-3ʹ; rev: 5ʹ-CCACGCGCCGATTTCCAAGG-3ʹ; 137 bp.

*IL-1B*: *Interleukin 1 Beta Proprotein*, *Catabolin*; NM_000576.2; fw: 5ʹ-CACGATGCACCTGTACGATCA-3ʹ; rev: 5ʹ-GTTGCTCCATATCCTGTCCCT-3ʹ; 121 bp.

*ITPK1*: *Inositol 1,3,4-Triphosphate 5/6 Kinase*; NM_014216.3; fw: 5ʹ-ACCCGCTCCCTGCCATCAGA-3ʹ; rev: 5ʹ-CGCATGGTGTCATCCCCGCA-3ʹ; 145 bp.

*TNF-alpha*: NM_000594.2; fw: 5ʹ-ATGAGCACTGAAAGCATGATCC-3ʹ; rev: 5ʹ-GAGGGCTGATTAGAGAGAGGTC-3ʹ; 217 bp.

*TRAF4*: *TNF Receptor-Associated Factor* 4; NM_004295.3; fw: 5ʹ-GTGATTATCCACGTTTCACC-3ʹ; rev: 5ʹ-CCCTGTGTAGCTCAGAAACT-3ʹ; 193 bp.

#### Genes-of-interest found not differentially expressed

*AP-1* (*JUN*): *Jun Proto-Oncogene*; NM_002228.3; fw: 5ʹ-GCACATCACCACCACGCCGA-3ʹ; rev: 5ʹ-CACCATGCCTGCCCCGTTGA-3ʹ; 172 bp.

*CARD4* (*NOD1*): *Nucleotide-Binding Oligomerization Domain Containing 1 (NOD1) or CARD4*; NM_006092.2; fw: 5ʹ-TCTGTGGAGATGCCGTTGGAC-3ʹ; rev: 5ʹ-TCGCCCTGGCTGTGA AGA AC-3ʹ; 154 bp.

*BAD*: *BCL2-Antagonist of Cell Death*; NM_032989.2 (transcript variant 1), NM_004322.3 (transcript variant 2); fw: 5ʹ-ACGAGTTTGTGGACTCCTTT-3ʹ; rev: 5ʹ-GTACTTCCGCCCATATTCA-3ʹ; 226 bp.

*COX2*: *Prostaglandin-Endoperoxide Synthase 2* (*Prostaglandin G/H Synthase* and *Cyclooxygenase*); NM_000963.2; fw: 5ʹ-TGCCTGGTCTGATGATGTATG-3ʹ; rev: 5ʹ-TTAGCCTGCTTGTCTGGAAC-3ʹ; 120 bp.

*CXCR4*: *Chemokine (C-X-C motif) Receptor* 4; NM_003467.2 (transcript variant 1), NM_001008540.1 (transcript variant 2); fw: 5ʹ-AGATGATGGAGTAGATGGTGGG-3ʹ; rev: 5ʹ-TACACCGAGGAAATGGCCTCA-3ʹ; 112 bp.

*DDX50*: *DEAD* *(Asp-Glu-Ala-Asp) box polypeptide 50*; NM_024045.1; fw: 5ʹ-GCCTTGCCCCCGCTTCCTTT-3ʹ; rev: 5ʹ-CCCCCAGAGGAGTTTCCCAGGC-3ʹ; 109 bp.

*E2F3*: *E2F Transcription Factor 3*; NM_001949.3; fw: 5ʹ-AACGCACAGTTGCAGGCTCC-3ʹ; rev: 5ʹ-AGCAAGCCAATCCGGGGAGGAA-3ʹ; 147 bp.

*E2F4*: *E2F Transcription Factor 4, p107/p130-binding*; NM_001950.3; fw: 5ʹ-AGCCCAGTCCCAGGAAGCCT-3ʹ; rev: 5ʹ-AGTGGCCGGGTGTCCAGTGT-3ʹ; 198 bp.

*E2F7*: *E2F Transcription Factor 7*; NM_203394.2; fw: 5ʹ-AGTCACGAAACACCAGCTCGGC-3ʹ; rev: 5ʹ-TTTGCATCCCGCCTCGGACA-3ʹ; 133 bp.

*E2F8*: *E2F Transcription Factor 8; NM_024680.2*; fw: 5ʹ-ACAGCACCGTCCCTCATCCA-3ʹ; rev: 5ʹ-TTGGTGGGCTTGAGTGGGCT-3ʹ; 141 bp.

*HMOX1*: *Heme Oxygenase (Decycling) 1*; NM_002133.2; fw: 5ʹ-GTTGAGCAGGAACGCAGTCTT-3ʹ; rev: 5ʹ-CAGTGCCACCAAGTTCAAGC-3ʹ; 112 bp.

*IRF2*: *Interferon Regulatory Factor 2*; NM_002199.3; fw: 5ʹ-GGCATGGCGTCCTTCGTCACTT-3ʹ; rev: 5ʹ-TGCTGGATGCTGGGGTCATGGA-3ʹ; 142 bp.

*PMYKT*: *protein kinase, membrane associated tyrosine/threonine (transcript variant 1)*; NM_004203; fw: 5ʹ-CCGCCACGCAGAACCTGGAT-3ʹ; rev: 5ʹ-GCGGCTGATGGGAATGCTGC-3ʹ; 100 bp.

*RB-1*: *Retinoblastoma 1*; NM_000321.2; fw: 5ʹ-GTCATGCCGCCCAAAACCCC-3ʹ; rev: 5ʹ-GCTGTCCTGCTCTGGGTCCT-3ʹ; 114 bp.

*TANK*: *TRAF family member-associated NFKB activator (transcript variant 2)*; NM_133484.1; fw: 5ʹ-ATTATGGCTGTGTTCCTCTG-3ʹ; rev: 5ʹ-GAAGCAATGTCTACCTTTGG-3ʹ; 127 bp.

*TP73*: *Tumor Protein p73*; NM_005427.2; NM_001126240.1, NM_001126241.1 NM_001126242.1 (transcript variant 1–3); fw: 5ʹ-AAGCTGCCCTCCGTCAACCA-3ʹ; rev: 5ʹ-TGCTCATCTCGCCGTTGGCT-3ʹ; 136 bp.

#### Reference Genes for normalization

*ACTB*: *Actin, beta*; NM_001101.3; fw: 5ʹ-GGCATGGGTCAGAAGGATT-3ʹ; rev: 5ʹ-AGGTGTGGTGCCAGATTTTC-3ʹ; 133 bp.

*GAPDH*: *Glyceraldehyd-3-Phosphat-dehydrogenase*; NM_002046.3; fw: 5ʹ-CTCTGCTCCTCCTGTTCGAC-3ʹ; rev: 5ʹ-CAATACGACCAAATCCGTTGAC-3ʹ; 116 bp.

*HPRT1*: *Hypoxanthine phosphoribosyl-transferase 1*; NM_000194.2; fw: 5ʹ-CCTGGCGTCGTGATTAGTGAT-3ʹ; rev: 5ʹ-AGACGTTCAGTCCTGTCCATAA-3ʹ; 131 bp.

*RPLP0*: *Ribosomal Protein, large, P0*; NM_001002.3; fw: 5ʹ-TGGCAATCCCTGACGCACCG-3ʹ; rev: 5ʹ-TGCCCATCAGCACCACAGCC-3ʹ; 194 bp.

*TUBB*: *Tubulin, beta*; NM_178014.2; fw: 5ʹ-TTGCCCCTCTCACCAGCCGT-3ʹ; rev: 5ʹ-CGGAAGACAGCAGCCACGGT-3ʹ; 125 bp.

Synthesis of ceramide analog synthetic compounds are given in Supplementary Material.
