# Supplementary material for: Stereospecific induction of apoptosis in tumor cells via endogenous C16-ceramide and distinct transcripts
Source: Cell Death Discov. 2015 Jul 27;1:15013–. doi: 10.1038/cddiscovery.2015.13 (PMC4979478; doi:10.1038/cddiscovery.2015.13)
Supplement: Supplementary Information [file cddiscovery201513-s1.pdf]

Supplemental material, data and statistics

Stereospecific induction of apoptosis in tumor cells via endogenous C_{16}-ceramide and distinct transcripts

M. Blaess[1,2], HP Le[3], RA Claus[1,2], M Kohl[3] and H-P Deigner[3,4*]

[1]Center for Sepsis Control and Care (CSCC), Jena University Hospital, Erlanger Allee 101, D-07747 Jena, Germany

[2]Clinic for Anaesthesiology and Intensive Care, Jena University Hospital, Erlanger Allee 101, D-07747 Jena, Germany

[3]Furtwangen University, Medical and Life Sciences Faculty, Jakob-Kienzle-Str. 17 D-78054 Villingen-Schwenningen, Germany

[4]Fraunhofer Institute IZI, Leipzig, EXIM Department, Schillingallee 68 D-18057 Rostock, Germany

*e-mail: hans-peter.deigner@hs-furtwangen.de

1 MIAME-file

Microarray experiment description file (according to the MIAME Checklist – update January 2005)[1].

1.1 Experimental designs and experimental details

Elucidation of single steps of apoptosis initiated by synthetic, metabolically stabilized analogs of ceramide with (partially) defined stereochemical configuration offers a valuable strategy, to define putative structure-activity relationships and may foster the development for the treatment of cancer or for enhancing or retaining the potency of well-known cancer drugs.

Keywords: Oligonucletid spotted Microarray, Human colon carcinoma cells HT-29, synthetic Ceramide analoga

Experimental factors: Human colon carcinoma cells HT-29 were stimulated 4, 6, 8 and 24h with 30 µM HPL-1R36N (**6**) or HPL-1S36N (**7**) to measure the gene expression response.

Experimental design: Cell culture samples from four points in time had been obtained. Total RNA was isolated from HT-29 cells using standard techniques (Qiagen, Hilden, Germany). cDNAs of HPL-1R36N (**6**) treated HT-29 cells were labeled with DY-647-S-NHS-dye and co-hybridized with DY-548-S-NHS-dye labeled cDNA obtained from HPL-1S36N (**7**) treated HT-29 cells.
Microarray hybridization was performed using the SIRS-Lab GmbH Lab-Arraytor® 60-1 bis 60-6 Combi Oligonucleotid-Chip, comprising 593 gene probes as well as 22 control probes.

File-Name (*.gpr)	Incubation time
2004-11_24_2DE_ch20_01_01	4h incubation
2004-11_24_3DE_ch20_15_01	6h incubation
2004-11_30_4DE_ch21_01_01	8h incubation
2004-11_30_5DE_ch21_20_01	24h incubation

Supplementary Table 1: Raw Data-Files of HPL-1R36N (**6**) / HPL-1S36N (**7**) treated HT-29 cells on Lab-Arraytor® 60-1 to 60-6 Combi Oligonucleotid-Chip, microarray analysis

Quality control steps taken: Quality control of isolated total RNA (18S / 28S peaks of ribosomal RNA) was checked using denaturateing gel elektrophorese (1.2% formaldehyde Agarose gel) and a Bioanalyzer® 2100 (Agilent Technologies). Further control steps: presence of repetitive elements (retroposons), unspecific binding, traces of spotting buffer, RNA isolation yield, possible degradation of one and / or both RNA specimens, reverse transcription processivity and efficiency and efficiency, dye coupling efficiency, hybridization uniformity, scanning output, spot finding and flagging.

Links to the publication, any supplementary websites or database accession numbers:
Not available, proprietary commercial product.

1.1.1 Samples used, extract preparation and labeling

The origin of each biological sample:
Organism (NCBI taxonomy): Human (Homo sapiens);
Cell type: colon adenocarcinoma *HT-29* (DSMZ No: ACC299)

Manipulation of biological samples and protocols used:

Growth conditions: Cells are cultivated in 175 cm^3 cell culture flasks with 50 ml of a cell culture medium containing 90% McCoy´s 5A medium, 10% FCS, 2 mM L-Glutamine, 50 units/ml Penicillin, 50 µg/ml Streptomycin.
Treatments: 4, 6, 8 and 24 h with 30 µM HPL-1R36N (**6**) or HPL-1S36N (**7**) semi-confluent in 5.8 cm^2 petri dishes at 37° C and 5% CO2 with McCoy´s 5A Medium containing 1% human AB serum, supplemented with 2mM L-Glutamine, 50 units/ml Penicillin, 50 µg/ml Streptomycin in an cell culture incubator.

Separation techniques: none

Experimental factor value for each experimental factor, for each sample: none

Technical protocols for preparing the hybridization extract:

Extraction method: Monolayers were washed with ice-cold PBS and cells were scraped in 3 ml lysis buffer and total RNA from HT-29 cells was extracted with RNeasy Mini Spin Columns (Qiagen, Hilden, Germany). RNA isolation was performed according to manufacturer's recommendations.
No amplification method was used.
Label used: monofunctional NHS-(succin-imidyl)-esters derivatives for both DY-648-S-NHS and DY-548-S-NHS.
Label incorporation method: cDNAs were cleaned with Microcon® YM-30 (Amicon) and labeled using Dyomics DY-648-S-NHS dye (HPL-1R36N (**6**) stimulated cells) and Dyomics DY-548-S-NHS dye (HPL-1S36N (**7**) stimulated cells). Unattached dye was removed with Promega Wizard® PCR clean-up kit (Promega) according to manufacturers instructions.

External controls (spikes), if used: none

1.1.2 Hybridization procedures and parameters

Information concerning concentration of solutes in the hybridization mixture and blocking agents used represents a confidential information property of SIRS-Lab GmbH, Jena (Germany).

Quantity of labeled target used: identical amounts of obtained labeled cDNA were used in each hybridization step and identical amounts of labeled cDNA (obtained from stimulated / unstimulated samples) were cohybridized.

Time, concentration, volume, temperature:

hybridization time: 10 h
concentration: whole amount of labeled amplified cDNAs
volume: 90 µl
hybridization temperature: 42 °C

Wash procedure: soaking of hybridization chamber with wash buffer I (2xSSC, 0.03%SDS) for 30 seconds; double washing in buffer I for 1.5 min at room temperature each, with soaking of hybridization chamber with wash buffer I for 30 seconds in between; soaking of hybridization chamber with wash buffer II (1xSSC) for 30 seconds;

double washing in buffer II for 1.5 min at room temperature each, with soaking of hybridization chamber with wash buffer II for 30 seconds in between; soaking of hybridization chamber with wash buffer III (0.2xSSC) for 30 seconds;

washing in buffer III for 1.5 min at room temperature; array surface drying by application of a nitrogen under pressure 2.5 bar for 2.5 min at 30°C.

Description of the hybridization instruments: The HS 400 Hybridization Station is a system that fully automates the process of hybridization of arrays spotted on microscopic slides and is part of Tecan's Array Suite (Tecan). It consists of a built-in Liquid Distribution Unit, a waste system and one module with a heating block and a chamber frame for securing the slide adapter with 4 slides, thus, it has the ability to perform hybridization with a maximum of 4 slides which can be manipulated simultaneously. The slides are temperature-controlled and can be heated and cooled between 4°C and 85°C. Vacuum-driven agitation of the hybridization mixture produces a homogeneous DNA concentration over array area and augments a slow diffusion by liquid movement along the long slide axis. This results in higher spot signals and homogeneous low background level over the whole slide. The station has ports for 6 reagent bottles, 4 of which are temperature-controlled. They are used for hosting and applying pre-hybridization, washing buffers etc. The On-Board slide drying allows the slides to be automatically dried by using pressurized nitrogen. The HS 400 Hybridization Station is controlled by an external PC with HS Control Manager Software.

Measurement data and specifications: does not apply

1.1.3 Data extraction and processing protocols

Scanning hardware : GenePix® 4000B confocal epifluorescent scanner (Axon Instruments)
Scanning software: GenPix® Pro 4.0
Scan parameters: Laser power: Cy3 channel – 100%; Cy5 channel – 100%
PMT voltage: Cy3 channel – 700 V; Cy5 channel – 800 V
Spatial resolution (pixel space): 10 µm.

Each RNA pair was labeled with two different dyes and cohybridised with the same microarray. The sample from HPL-1R36N (**6**) stimulated cells was labeled with Dyomics DY-648-S-NHS dye. In turn, the RNA isolated from HPL-1S36N (**7**) stimulated cells was labeled with Dyomics DY-548-S-NHS dye. Digital images resulting from posthybridization array scanning were quantified using GenePix® Pro 4.0 software (Axon Instruments). For the (**i**) spot detection and quantification and (**ii**) spot quality flagging, the GenePix® Analysis Software was used. The raw expression signals for each spot were quantified as the median spot intensity in the red and green channel, corrected by the median local background intensity. The spots were flagged corresponding to the settings of the GenePix® Software (100 = "good", 1 = "weak /saturation", 0 = "not found", -100 = "bad"). The raw signals of a microarray were summarised in *gpr files according to supplemental table 1.

1.1.4 Normalization, transformation and data selection procedures and parameters

Normalization and transformation: For the normalization and a variance stabilised transformation of raw signals, the approach of Huber[2,3] was employed, where the additive and multiplicative errors were estimated, block by block, from ca. 70% of all spots. The normalised signals were transformed by *asinh*[1].

Filtering: The technical replicates on a microarray (multiple spots of the same probe) were filtered from the corrected and transformed signal intensities depending on their spot quality. Per spot, replicates with the highest flag value were selected and the corresponding signal intensities were averaged. Expression of spots with only invalid replicates was labeled by NaN (not a number). The quality of resulting gene expression was flagged by:
100 / 66 / 33 relative frequency of „good“ replicates, if some "good" replicates occurred; **1 / 0.6 / 0.3** relative frequency of „weak“ replicates, if no "good" but some "weak" replicates occurred, **0**, if no replicate was flagged better than “not found“; **-100**, if all replicates were flagged by "bad".

The normalized and transformed data were summarized in the Excel file *datamatrix_HPL_1RS36N.xls.*

[1] The data were transformed by the arcsinh function, e.g., the transformed ratio of ± 0.4 correspond to 1.5 fold change (almost identical with the natural logarithm), the transformed intensity of 5 correspond to ca. 75 random fluorescence units [rfu] ((almost identical with the half of the natural logarithm).

1.1.5 Array Design

1.1.5.1 General array design, platform type

Array design name: Lab-Arraytor® 60-1 bis 60-6 Combi Oligonucleotid-Chip, SIRS-Lab GmbH, Jena (Germany)

Platform type: spotted microarrays.

Surface and coating specification: Epoxysilane coated glass slides for covalent immobilization of amino-modified DNA or polynucleotides. The surface-modified slides Typ E were developed at Schott-Nexterion. (Schott Group, Germany)

Physical dimensions of array support: 26 x 76 x 1 mm microscope glass slide.

Number of features on the array: The microarray consists of 593 well-characterized human genes and a set of 22 standard control features. The spots are organized into 4 blocks of 4 identical subarrays. Each subarray is spotted in a block of 14 by 14 spots

Availability: The aforementioned microarray is available for purchase.

Spot dimensions: diameter 120 ± 10 µm. Spotting pattern: 14 x 14 spots using 28 SMP 4 Spilt pins (Telechem Inc. USA)

Attachment chemistry: covalent

1.1.5.2 Array feature and reporter annotation

Type of reporter: synthetic polynucleotides, 56-70 deoxynucleotide residues long; single-stranded.

Reporter sequence: Sequence or PCR primer information: all information concerning the polynucleotide sequences used for array elements generation represents a confidential property of SIRS-Lab GmbH (Jena, Germay).

Sequence information: all information concerning gene symbols and Genebank accession numbers used for the polynucleotides sequence design are included in the corresponding raw data file: Oligonucleotide array description file.xls).

Composite sequence: Polynucleotide probes for different regions of 3'-part of human β-actin gene and glyceraldehyde-3-phosphate dehydrogenase gene located on the distance of approximately 200 bases along their cognate mRNA sequences.

1.1.5.3 Control elements on the array:

Positive control elements: spots of probes for different regions of 3'-part of human β-actin gene, glyceraldehyde-3-phosphate dehydrogenase gene and genes for two isoforms of human tubulin alpha.

Negative control elements: blank spots; spotting buffer spots.

Control elements with specialized functions: spots containing polynucleotide probes for 12 annotated human genes known to maintain their RNA expression rate at constant level in HT-29 cells undergoing HPL-1R36N (**6**) or HPL-1S36N (**7**) stimulation; spots and containing polynucleotide probes for 2 annotated human genes known to maintain their RNA expression rate at constant level in most in vitro and in vivo investigated

biological systems and their responses to perturbed environment (“house-keeping” genes).

1.1.5.4 Feature location on the array:

All information concerning the location of the reporters on the microarray are included in the raw data file: **Oligonucleotide array description file.xls**
All information concerning the characteristics of the reporter molecule on the microarray are included in the raw data file: **Oligonucleotide array description file.xls**
All information concerning the biological annotation of each of the reporter molecules on the microarray are included in the raw data file: **Oligonucleotide array description file.xls**

Principle array organism(s): Human sequence probes

1.2 Statistical methods

Statistical evaluation of the data is performed using statistical software R[4]. It starts with the definition of two meaningful and sufficient parameters to classify a gene significantly changed in his expression at a selected timepoint compared to a reference sample.
The two parameters are **a**) the limit where a significant change in gene expression can be accepted and **b**) denotes the minimum value of the mean signal intensity, where the fluorescence signal of the hybridized cDNA from the background fluorescence is significantly distinguished (also known as "solid spot quality"). A significant change in the gene expression, is assumed if the absolute amount of ln change is greater than 0.4 (signals differ more than 50% to each other), and the spots were flagged with a non-negative value. Therefore, only spots are included into statistical analysis having been classified as "found / not flagged" (1) or "good" (100).

1.3 Diagnostic plots - array / hybridization quality control plots

In addition to the normalization of the data, some diagnostic plots (boxplots of log-ratios, mean intensities of the normalized data, and a mean-sd-plot) are generated. In Supplementary Figure 1 the boxplots of the log-ratios, the mean intensities of the normalized data and the Mean-SD plot are presented (Supplementary Figure 1a-d). These plots indicate that the correction of the systematic error and the variance stabilizing transformation works well. No abnormalities were found.

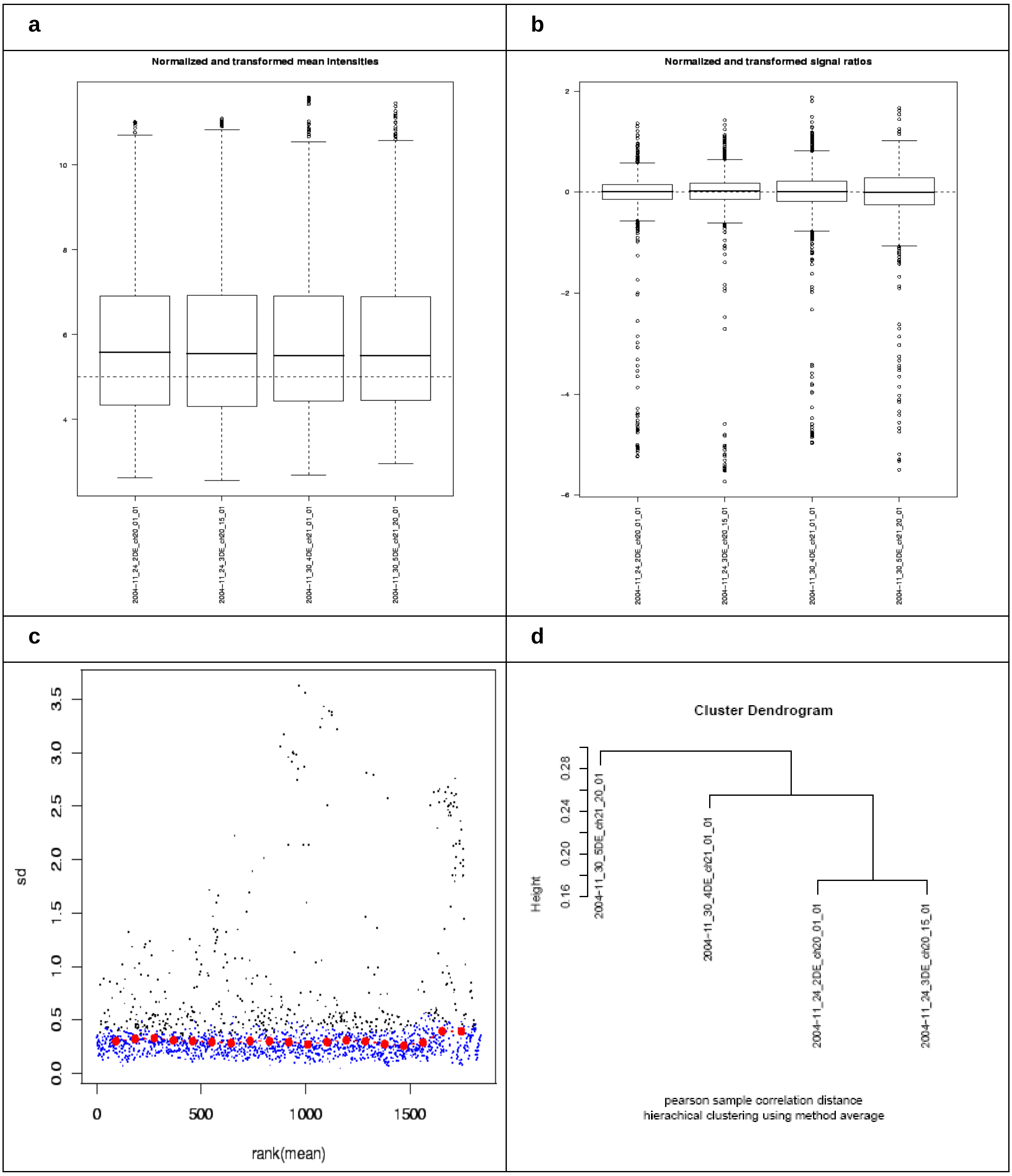

Supplementary Figure 1: (**a**) Boxplots of mean intensities, (**b**) normalized and trandformed signal log-ratios, (**c**) mean-SD-Plot of normalized data and (**d**) clustering dendrogramm (hierarchical clustering of arrays using average linkage method and Pearson's sample correlation).

1.4 Effects of HPL-1R36N (6) and HPL 1S36N (7) on gene expression in HT-29 cells

1.4.1 Considering all time points

To get an overview of all effects occurring, statistical analysis starts appropriately with the consideration of all investigated parameters (incubationtime / experimental conditions). By applying parameters for statistically significant changes in genes on the total of 593 genes addressed on the chip, the result is 158 gene probes, which have sufficient signal intensity and are classified differentiated expressed at at least one time point. They correspond to 151 genes that are marked as "selected spots" and colored dark purple in Supplementary Figure 2a. The remaining gene probes either have low signal intensity or show no significant effects in the considered time frame.

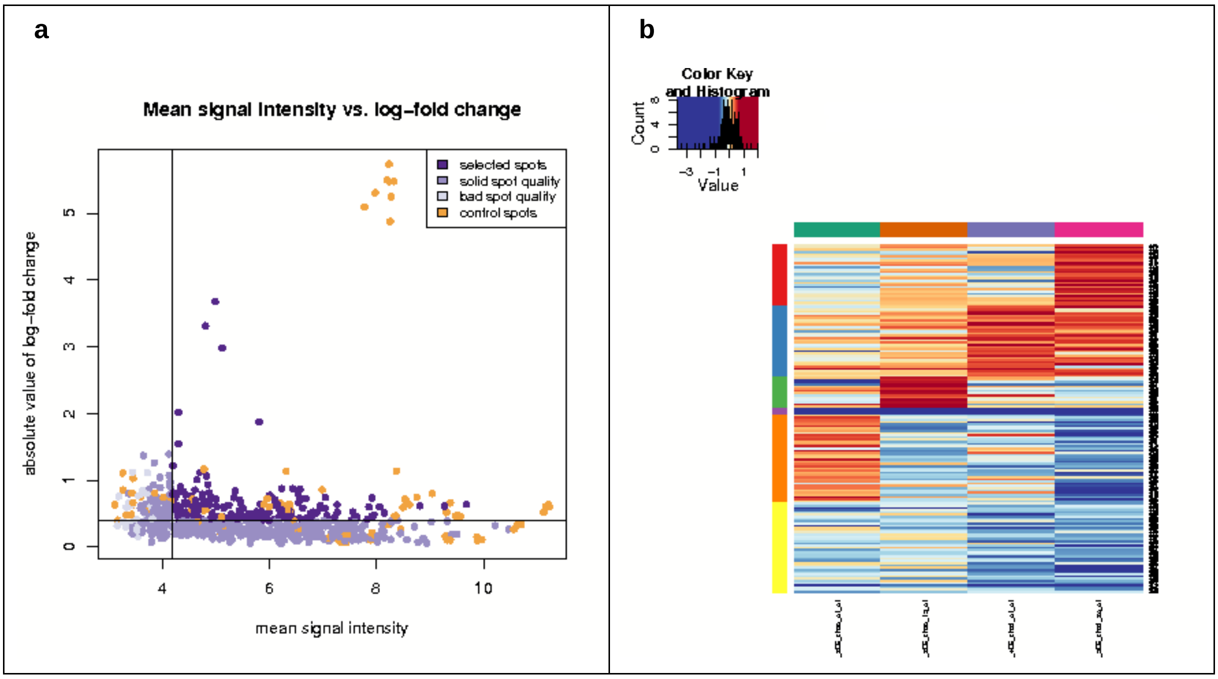

Supplementary Figure 2: (**a**) Plot of the average signal intensity against the absolute value of the log-fold change in the amount of change in the expression level to the base 2 (ln). (**b**) Heat map of expressed genes differentiated at least one time. Red-colored genes are regulated by HPL-1R36N (**6**) compared to HPL-1S36N (**7**) up, blue-colored genes down. The incubation period is starting from the left 4, 6, 8 and 24 h (corresponding slides 2D/E, 3D/E, 4D/E, 5D/E). Similarly behaving genes are grouped into clusters, which are marked by a colored bar (in the heat map on the left).

1.4.2 Consideration individual time points

The six clusters of the heatmap in Supplementary Figure 2b are mainly defined by genes that behave similarly over the entire observation period. Four of these clusters are defined by genes (4, 6, 8 or 24 h) that are expressed maximally differentiated at a certain point of time. To gain an overview of the behavior of these genes at each point of time during incubation period, a time course heat map of these differentially expressed and selected genes is created.

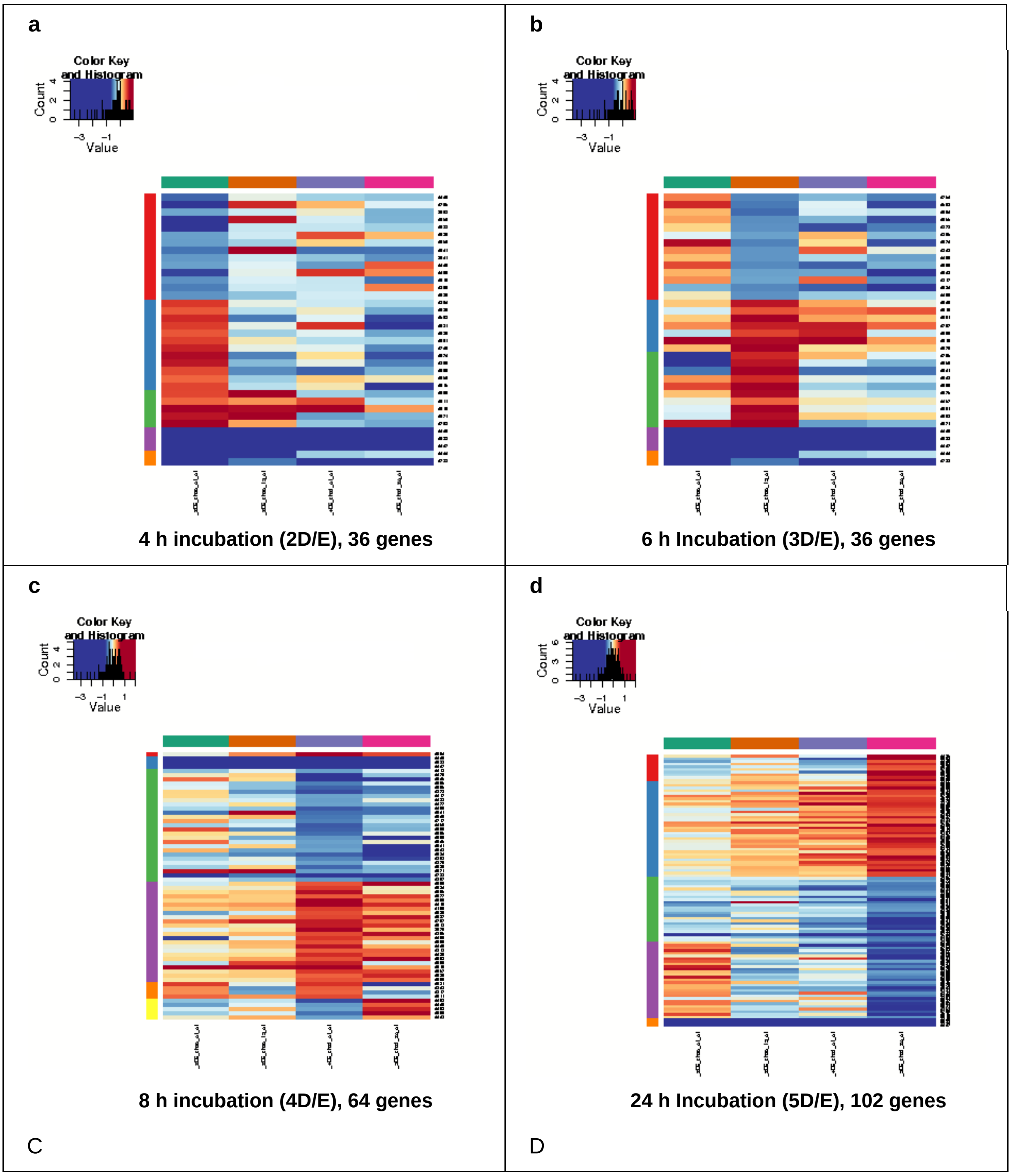

Supplementary Figure 3: Heat maps of differentiated the expressed genes after 4 h (**a**), 6 h (**b**), 8 h (**c**) and 24h (**d**) and their time course. Incubation period is starting from the left 4, 6, 8 and 24 h (corresponding slides 2D/E, 3D/E, 4D/E, 5D/E). Red-colored genes are up regulated by HPL-1R36N (**6**) compared to HPL-1S36N (**7**), blue-colored genes are down-regulated. Supplementary Figure 3d may not be confused with Supplementary Figure 3b, since it contains genes that are **at least** differentially expressed at time point 24 h.

1.5 Differentially expressed genes in the hybridization experiment and real-time PCR

1.5.1 Overview up regulated genes

RefSeq	Symbol	Flag	incubation [h]				maximum
			4 h	6 h	8 h	24 h	
NM_031908.4	C1QTNF2	1/100	-1,042	1,369	7,506	1,616	7,506
NM_139239.1	TA-NFKBH	1	-1,315	-1,109	-1,722	4,700	4,700
NM_183395.1	CIAS1	1	1,337	1,340	1,209	3,390	3,390
NM_001565.2	CXCL10	1/100	2,542	1,923	2,418	1,320	2,418
NM_006609.3	MAP3K2	1	-1,713	2,460	-1,738	-1,729	2,460
NM_004295.1	TRAF4	100	-1,153	1,213	1,174	2,396	2,396
NM_004295.1	TRAF4	100	-1,520	-1,078	-1,538	1,532	1,532
NM_004322.2	BAD	100	1,023	1,439	-1,086	2,396	2,396
NM_000619.2	IFNgamma	1	1,919	-1,657	1,073	-1,871	1,919
NM_002985.2	CCL5	1	1,494	1,335	1,598	-1,205	1,598

Supplementary Table 2: Time course of in the hybridization experiment most highly regulated genes. The table is complemented by genes CCL5 and IFNγ that are regulative in context of CXCL10. To estimate the quality (flag) of the spots, flagging values of distinct spots after 4 h incubation (2 D/E) are given as an example.

1.5.2 Overview on down-regulated genes

RefSeq	Symbol	Flag	incubation [h]				maximum
			4 h	6 h	8 h	24 h	
NM_002521.2	NPPB	1	-39,398	-11,018	-8,320	-3,573	-39,398
NM_003841.2	TNFRSF10C	1	-27,457	-5,529	-2,899	-3,763	-27,457
NM_006092.1	CARD4	1	-19,705	-2,769	-3,753	-2,626	-19,705
NM_001952.3	E2F6	100	-6,479	-2,852	-1,304	-1,205	-6,479
NM_198256.1							
NM_006684.2	CFHR4	1	-2,207	-1,705	-3,062	-2,945	-2,945
NM_001242.3	TNFRSF7	1	-1,202	1,076	-1,209	-2,894	-2,894
NM_024110.2	CARD14	1	-2,737	1,755	1,214	-1,090	-2,737
NM_002199.2	IRF2	100	-2,372	1,868	-1,129	-1,433	-2,372
NM_177924.2							
NM_004315.3	ASAH1	1	1,002	1,129	-2,299	-1,354	-2,299
NM_133484.1	TANK	100	-1,276	-1,050	-1,637	-1,937	-1,937
NM_002089.1	CXCL2	1	-1,304	1,133	-1,776	-1,448	-1,776
NM_002228.3	JUN / AP-1	1	-1,112	-1,021	-1,213	-1,647	-1,647
NM_207002.1	BCL2L11	100	1,390	-1,091	-1,221	-1,668	-1,668

Supplementary Table 3: Time course of in the hybridization experiment most down regulated genes. The table is complemented by CXCL2, AP-1, and BCL2L11, genes that play a role in apoptosis / cell death.

1.5.3 Comparison results hybridization experiment - real-time PCR

	real-time PCR					spotted oligonucleotid-array			
Symbol	2 h	4 h	6 h	8 h	24 h	4 h	6 h	8 h	24 h
CXCL10	-1,405	5,454	2,470	2,776	-1,671	2,542	1,923	2,418	1,320
CCL5	-1.048	2,333	1,238	1,828	-1,521	1,494	1,335	1,598	-1,205
TNFalpha	-1,114	2,343	2,468	3,834	-4,264	n.diff.exp.	n.diff.exp.	n.diff.exp.	n.diff.exp.
IFNgamma	n.exp.	n.exp.	n.exp.	n.exp.	n.exp.	1,919	-1,657	1,073	-1,871
TRAF4	-1,168	-1,257	1,302	1,056	-2,021	-1,153	1,213	1,174	2,396
						-1,520	-1,078	-1,538	1,532
ASAH1	1,063	-1,257	-1.018	1.012	-2,252	1,002	1,129	-2,299	-1,354
CARD4	-1,173	1,093	-1.077	1,053	-1,271	-19,705	-2,769	-3,753	-2,626
BCL2L11	1,040	1,101	1,004	1.056	-1,894	1,390	-1,091	-1,221	-1,668
IL1beta	-1,094	-1,034	1.026	1,492	-1,663	n.diff.exp.	n.diff.exp.	n.diff.exp.	n.diff.exp.
CXCR4	-1.146	1,241	-1,130	-1,154	-1,546	n.diff.exp.	n.diff.exp.	n.diff.exp.	n.diff.exp.

Supplementary Table 4: Comparison of the ratio of the relative expression level of HPL-1R36N (**6**) / HPL 1S36N (**7**) (Fold-ratio) of both methods in non-logarithmic representation. The numerical values of the down-regulated genes are transformed in the negative inverse ratio of the relative expression level HPL 1R36N (**6**) / HPL 1S36N (**7**). Abbreviations used: undifferentiated expressed - (n.diff.exp); no detectable expression (n.exp.). 2 h samples were not hybridized to the chip.

Quantitative real-time PCR has been performed on a BioRad i-Cycler® iQ. Two independent probes of TRAF4 are spotted on the Lab Arraytor® 60-1 to 60-6 cardio-combi-oligonucleotide array; therefore, two readings are listed. IL1beta, CXCR4 and TNFalpha (NM_000594) were chosen regarding the biological context. In the hybridization experiment, the genes have been unremarkable. IFNgamma has been remarkable in the hybridization experiment, allthough in real-time RCR expression could not be confirmed. COX2, HMOX1 are among the genes that remained unchanged in both methods, they are not listed in the table.

2 Synthesis of ceramide analog synthetic compounds

2.1 HPL-1RS36N (8) ((1RS)-(E)-1-(2-Methyl-oxazol-4-yl)-hexadec-2-en-1-ol)

2.1.1 4-Formyl-2-methyl–oxazol (9):

To a solution of 1.0 g (6:45 mmol) 2-methyl-oxazole-4-carboxylic acid ethyl ester in 50 ml anhydrous toluene a 9.86 mmol DIBAH solution at -60 °C is added over under argon atmosphere and stirred 40 minutes at -60 °C[5]. 15 ml of a mixture of saturated potassium sodium tartrate solution and ethyl acetate (1:2) are added to the flask and stirred for another 30 minutes. The flask content is shaken with a mixture of ethyl acetate (3x 30 ml) and saturated potassium sodium tartrate solution (3x10 ml). Pooled organic phases are dried over MgSO4. After filtration and removal of the solvent, the residue is purified by column chromatography (silica gel, ethyl acetate / n-hexane 1: 2 (V/V)). 400.0 mg (3.6 mmol, 55.9%) of the product is obtained.

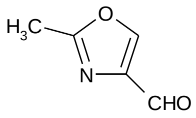

(**9**)

Supplementary Figure 4: 4-Formyl-2-methyl–oxazol (**9**).

IR (KBr): 3028 (w), 2924/2854 (m), 1710 (m), 1682 (s), 1597 (w) cm^{-1};
MS (80eV, 20°C): m/z (%) = 112 (14) $[M+1]^+$, 111 (62) $[M]^+$, 83 (12), 42 (100);
^{1}H NMR (360 MHz, $CDCl_3$, FN:LEH 120): δ (ppm) = 10.0 (m, C*H*O), 7.6 (s, C=C*H*O), 2.4 (s, C*H*$_3$);
^{13}C-NMR (90.58 MHz, $CDCl_3$, FN:LEC 120): δ (ppm) = 198.2 (s, *C*HO), 162 (s, O*C*N), 154.1 (s, N*C*=CO), 136.7 (s, C=*C*HO), 14.9 (s, *C*H$_3$);
HR-MS (80eV, 20°C): $[M]^+$ calc. for $C_5H_5NO_2$: 111.0320; found: 111.0325; analysis C5H_5NO_2 (111.10): calcd. ,found C(54.06,54.01), H(4,54,4,50), N(12.61,12,55), O(28,80,28,94).

2.1.2 HPL-1RS36N (1RS)-(E)-1-(2-Methyl-oxazol-4-yl)-hexadec-2-en-1-ol (8) and (2RS)-(E,Z)-Heptadec-3-en-2-ol (10)

To a solution of 0.25 g (1.2 mmol) pentadecin in 10 ml anhydrous n-hexane 1.4 mmol toluene-DIBAH solution is added under argon atmosphere and heated 2 h at 60 °C. The flask content is first cooled to 10 °C then a solution of 0.13 g (1.2 mmol) 4-formyl-2-methyl-oxazole (**9**) in 12 ml of a mixture of anhydrous toluene / diethyl ether (1:2) is added dropwise and stirring continued for another 1 h at RT. Then 15 ml of a mixture of ethyl acetate and saturated potassium sodium tartrate solution (2:1) is

added to the flask and stirred for another 30 minutes. The flask contents are shaken first with ethylacetate (3x 50 ml) then with saturated potassium sodium tartrate solution (3x 10 ml). The combined organic phases are dried over $MgSO_4$. After fitration and removal of the solvent, the residue is purified by column chromatography (silica gel, ethylacetate / n-hexane 1:2 (V/V)). Two products a) (identified as HPL-1RS36N (**8**)): 60 mg (0.187 mmol, 15.6%, Rf = 0:36), b) (identified as (2RS)-(E,Z)-Heptadec-3-en-2-ol (**10**)): 100 mg (0.67 mmol, 55.8%, Rf = 0.76) were isolated.

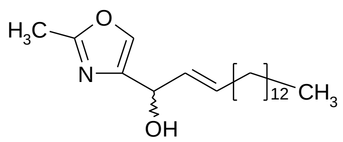

(**8**)

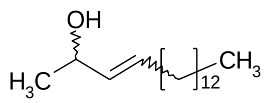

(**10**)

Supplementary Figure 5: Structures of HPL-1RS36N (1RS)-(E)-(2-Methyl-oxazol-4-yl)-hexadec-2- en-1-ol (**8**) and side-product (2RS)-(E,Z)-Heptadec-3-en-2-ol (**11**).

2.1.3 HPL-1RS36N (1RS)-(E)-(2-Methyl-oxazol-4-yl)-hexadec-2-en-1-ol (8):

IR (NaCl, Film): 3350 (s), 2924/2850 (s), 1716 (m), 1580 (w) cm^{-1};
MS (80eV, 76°C): m/z (%) = 322 (5) $[M+1]^+$, 321 (23) $[M]^+$, 138 (100), 112 (12), 82 (10), 41 (26); ^1H NMR (250.13 MHz, $CDCl_3$, FN:LEH 567): δ (ppm) = 7.4 (s, 1H), 5.8 (dd, ^3J = 15.7 Hz, ^3J = 7.1Hz, $CH=CH-CH_2$), 5.7 (dd, ^3J = 15.7 Hz, ^3J = 5.7 Hz, HOCH-CH=CH-), 5.1 (d, ^3J = 5.7 Hz, HOCH), 2.4 (s, $-CH_3$), 15.0-0.8 (m, $(CH_2)_{12}-CH_3$);
^{13}C-NMR (62.98 MHz, $CDCl_3$, FN:LEC 567): δ (ppm) = 162.2 (s, OCN), 143.0 (s, NC=CHO), 134.5 (s, CH=CH), 134.0 (s, NC=CHO), 128.4 (s, CH=CH), 78.0 (s, HOCH-), 32.1-22.5 (m, $(CH_2)_{12}-CH_3$), 14.1 (d, $2xCH_3$);
HR-MS (80eV, 20°C): $[M]^+$ calcd. for $C_{20}H_{35}NO_2$, 321.51; found: 321.4987; analysis $C_{20}H_{35}NO_2$ (321.51): calcd., found C(74.72,74.5), H(10.97,10.97), N(4.36,4.22).

2.1.4 (2RS)-(E,Z)-Heptadec-3-en-2-ol (10):

IR (NaCl, Film): 3450 (s), 2995/2985 (s),1500 (w) cm^{-1};
MS (80eV, 20°C): m/z (%) = 254 (5) $[M]^+$, 71 (36), 43 (49);
^1H NMR (360 MHz, $CDCl_3$, FN:LEH 598): δ (ppm) = 5.7 (m, CH=C*H*), 5.6 (m, C*H*=CH), 2.0 (m, HOC*H*), 1.4-0.8 (m, ^3J = 30 Hz, CH_3, (CH_2)$_{12}$-CH_3);
^{13}C-NMR (90.56 MHz, $CDCl_3$, FN:LEC 598): δ (ppm) = 137.8 (s, CH=*C*H), 127.4 (s, *C*H=CH), 32.8 (s, CH=CH-*C*H_2), 29.8 (s, HO*C*H), 29.7-29.1 (s, (*C*H_2)$_{10}$-CH_2-CH_3), 22.7 (s, *C*H_2-CH_3), 14.3 (s, 2x*C*H_3); HR-MS (80eV, 20°C): $[M]^+$ calcd. for $C_{17}H_{34}O$, 254.4610; found: 254.4615; analysis $C_{17}H_{34}O$ (254.46): calcd., found C(80.24,80.22), H(13.47,13,50), O(6.29, 6.28).

2.2 HPL-38N (4-[(1RS)-(E)-1-Hydroxy-3-phenyl-allyl]-(2RS,4R)-2-phenyl-thiazolidin-3-carbonic acid-tert-butyl-ester (4) and HPL-39N 4-[(1R)-(E)-1-Hydroxy-3-phenyl-allyl]-(2RS,4R)-2-phenyl-thiazolidin-3-carbonic acid-tert-butylester (3)

2.2.1 (2RS,4R)-4-Formyl-2-phenyl-thiazolidin-3-carbonsäure-tert-butylester (11)

2.2 g (6.8 mmol) (2RS,4R)-2-Phenyl-thiazolidine-3,4-dicarbonic acid-3-tert-butyl-ester-4-methylester is dissolved in 50 ml dry toluene, cooled to -70 °C; 13.6 mmol toluene-DIBAH solution is added dropwise and the mixture stirred 45 minutes at -50 °C. 15 ml of a mixture of ethyl acetate and saturated potassium sodium tartrate solution (2:1) is added to the flask and slowly warmed to RT. The flask content is extracted three times with a mixture of ethyl acetate (3x 50 ml) and saturated potassium sodium tartrate solution (3x10 ml) and the pooled organic phases dried over MgSO4. After removal of the solvent, the product is purified by column chromatography (silica gel, ethyl acetate:n-hexane (1:4) (V/V) as mobile phase. R_f = 0:36, 1:57 g, 5:36 mmol, 78.8%.

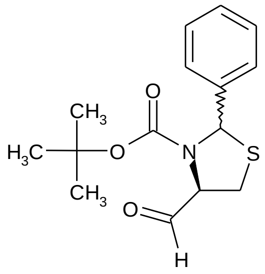

(**11**)

Supplementary Figure 6:(2RS,4R)-4-Formyl-2-phenyl-thiazolidin-3-carbonic acid-tert-butylester (**11**).

IR (NaCl, Film): 3050 (w), 2980 (m), 2910/2890 (m), 1701 (s), 1601 (m) cm^{-1};
MS (80eV, 67°C): m/z (%) = 293 (0.2) [M]+, 264 (14), 208 (3), 164 (54), 132 (4), 121 (7), 91 (10), 77 (4), 65(1), 51 (2), 41 (18);
^{1}H-NMR (360 MHz, $CDCl_3$, FN:LEH. 576): δ(ppm) = 9.8 (m, CHO), 7.45-7.2 (m, 5H), 6.0 (s, NCHS), 4.8 (m, $NCHCH_2$), 3.25 (m, $NCHCH_2S$), 1.5-1.1 (m, $3xCH_3$);
^{13}C-NMR (90.56 MHz, $CDCl_3$, FN:LEC: 576): δ(ppm) = 198.9 (s, CHO), 155.5 (s, OCON), 140.7 (s, Carom), 128 (s, 2xCH), 127.7 (s, 2xCH), 126.5(s, CH),77.4 (s, $C(CH_3)_3$), 69.0 (s, NCHS), 66.2 (s, OHCCHN), 32 (s, $NCHCH_2S$), 28.1 (s, $3xCH_3$); HR-MS (80eV, 20°C):
$[M]^+$ calcd. for $C_{15}H_{19}NO_3S$, 293.39;found:293,3895; analysis $C_{20}H_{35}NO_2$ (293.38): calcd., found C(61.41, 60.91), H(6.53, 6.64), N(4.77, 4.60).

2.2.2 HPL-38N 4-[(1RS)-(E)-1-Hydroxy-3-phenyl-allyl]-(2RS,4R)-2-phenyl-thiazolidin-3-carbonic acid-tert-butyl-ester (9) and HPL-39N 4-[(1R)-(E)-1-Hydroxy-3-phenyl-allyl]-(2RS,4R)-2-phenyl-thiazolidin-3-carbonic acid-tert-butylester (3)

To a solution of 500 mg (4.9 mmol) of phenylacetylene in 10 mL anhydrous n-hexane 5.0 mmol of DIBAH toluene solution is added and the mixture heated under reflux for 2 h. The flask contents are then cooled to 10 °C. 1.43 g (4.9 mmol) of (2RS, 4R)-N-tert-butoxycarbonyl-4-formyl-2phenyl-thiazolidine (**12**) is dissolved in 12 ml of a mixture of anhydrous toluene / diethyl ether (1: 2) and added dropwise at 0 °C. This mixture is stirred at RT for 1 h. 5 ml of ethyl acetate and 5 ml of saturated potassium sodium tartrate solution are added and stirring continued for another 30 minutes. The flask content is extracted three times with ethyl acetate (3x50 ml) and three times with saturated potassium sodium tartrate-solution (3x10 ml). After combining, the organic solvents are dried over magnesium sulphate, filtrated and the solvent is removed. The residue is loaded on a column and components eluted with ethyl acetate / n-hexane (1:4 V/V). Two products (9): 625.0 mg 1.57 mmol, 32%, (3): 875.0 mg, 2.2 mmol, 44.9 %) can be isolated.

2.2.3 HPL-38N 4-[(1RS)-(E)-1-Hydroxy-3-phenyl-allyl]-(2RS,4R)-2-phenyl-thiazolidin-3-carbonic acid-tert-butyl-ester (4):

IR (NaCl, Film): 3431 (s), 3028 (w), 2977/2929 (s), 1698 (s), 1598 (m) cm^{-1};
MS (80eV, 126°C): m/z (%) = 397 (0.02) $[M]^+$, 339 (1), 264 (19), 208 (7), 164 (81), 132 (3), 57 (100), 41 (12);
^{1}H-NMR (360 MHz, $CDCl_3$, FN:LEH. 604): δ(ppm) = 7.5-7.2 (m, 10*H*), 6.65 (d, ^3J =15.87 Hz, CH=C*H*), 6.2 (dd, ^3J = 15.87 Hz, ^3J = 5.8 Hz, CH=CH), 6.08 (s, NCHS), 4.6 (m, HOCH), 4.5 (m, NCH), 2.9 (m, $NCHCH_2$), 1.3-1.1 (m , $3xCH_3$);
^{13}C-NMR (90.56 MHz, $CDCl_3$, FN:LEC 604): δ(ppm)= 156.4 (s, NCO), 142.2 (s, Carom) 138.4 (s, Carom), 133.2 (s, CH=CH), 129.5 (s, 4xCH), 128.4 (s, CH=CH), 126.5 (s, 4xCH), 126.1 (s, 2xCH), 83.1 (s, $C(CH_3)_3$), 77.5 (s, HOCH-CH), 67.0 (s, NCHS), 66.5 (s, $NCHCH_2$), 33.0 (s, CH_2S), 28.0 (s, $3xCH_3$);
HR-MS (80eV, 20°C): $[M]^+$ calcd. for $C_{23}H_{27}NO_3S$ 397.54; found 397.536; analysis $C_{23}H_{27}NO_3S$ (397.536): calcd.,found C(69.49,69.14), H(6.84,6.83), N(3.52,3.31).

2.2.4 HPL-39N 4-[(1R)-(E)-1-Hydroxy-3-phenyl-allyl]-(2RS,4R)-2-phenyl-thiazolidin-3-carbonic acid-tert-butylester (3):

IR (NaCl, Film): 3408 (s), 3038 (w), 2977/2939 (s), 1698 (s), 1595 (m) cm^{-1};
MS (80eV, 126°C): m/z (%) = 397 (0.02) $[M]^+$, 339 (1), 264 (21), 208 (9), 164 (83), 132 (3), 57 (100), 41 (12).
^{1}H-NMR (360 MHz, $CDCl_3$, FN:LEH. 604): δ(ppm) = 7.5-7.2 (m, 10*H*), 6.65 (d, ^3J =15.9 Hz, CH=C*H*), 6.2 (dd, ^3J = 15.9 Hz, ^3J = 5.8 Hz, C*H*=CH), 6.1 (s, NC*H*S), 4.6 (m, HOC*H*), 4.6 (m, NC*H*), 3.0 (m, $NCHCH_2$), 1.3-1.1 (m , $3xCH_3$).
^{13}C-NMR (90.56 MHz, $CDCl_3$, FN:LEC 604): δ(ppm)= 156.6 (s, N*C*O), 142.5 (s, C_{arom}) 138.7 (s, C_{arom}), 133.3 (s, CH=*C*H), 129.6 (s, 4xCH), 128.7 (s, CH=CH), 126.9 (s, 4x*C*H), 126.1(s, 2xCH), 83.4(s, $C(CH_3)_3$), 77.5 (s, HOCH-CH), 67.2 (s, NCHS), 66.5 (s, $NCHCH_2$), 33.3 (s, CH_2S), 28.5 (s, $3xCH_3$);
HR-MS (80eV, 20°C): $[M]^+$ calcd. for $C_{23}H_{27}NO_3S$ 397.54; found 397.534; analysis: $C_{23}H_{27}NO_3S$ (397.534): calcd.,found C(69.49,69.24), H(6.84,6.73), N(3.52,3.41).

2.3 Separation HPL-1R36N (6), HPL-1S36N (7).

Chiral phase HPLC separation of HPL-1RS36N (**8**) was performed on a (R)-(-)-N-3,5-dinitrobenzoylphenylglycine (DNBPG) bonded chemically to silica gel (Bakerbond®, Regis Hi-Chro®) column with n-hexane-ethyl acetate-isopropanol (100:15:2 (v/v)) as mobile phase (flow: 0.5 ml/min) and UV detection at 260 nm affording the (S)-enantiomer (HPL-1S36N (**7**)) as first compound (RV 18.5 ml) followed by the (R)-enantiomer (HPL-1R36N (6)) (RV 19.4 ml)[6].

3 References

1. Brazma A, Hingamp P, Quackenbush J, Sherlock G, Spellman P, Stoeckert C et. al. Minimum information about a microarray experiment (MIAME)-toward standards for microarray data. Nature Genetics 2001: 29; 365–371.

2. Huber W, von Heydebreck A, Sültmann H, Poustka A, Vingron M. Variance stabilization applied to microarray data calibration and to the quantification of differential expression. Bioinformatics 2002; 18: 96-104.

3. Huber W, von Heydebreck A, Sültmann H, Poustka A, Vingron M. Stat. Parameter estimation for the calibration and variance stabilization of microarray data. *Appl. Genet. Mol.* 2003; **2:** Article 3.

4. R Development Core Team. *R: A Language and Environment for Statistical Computing* (R Foundation for Statistical Computing, Vienna, Austria, 2014). ISBN 3-900051-07-0, URL http://www.R-project.org.

5. Cornforth, JW, Cornforth, RH. A New Synthesis of Oxazoles and Iminazoles including its Application to the preparation of Oxazole *J. Chem. Soc.* 1947; 96-102.

6. Kühn H, Wiesner R, Lankin VZ, Nekrasov A, Alder L, Schewe T. Analysis of the stereochemistry of lipoxygenase-derived hydroxypolyenoic fatty acids by means of chiral phase high-pressure liquid chromatography. Anal. Biochem. 1987; 160; 24-34.
